# A nitrogen isotopic shift in fish otolith–bound organic matter during the Late Cretaceous

**DOI:** 10.1073/pnas.2322863121

**Published:** 2024-07-29

**Authors:** Zixuan C. Rao, Jessica A. Lueders-Dumont, Gary L. Stringer, Yeongjun Ryu, Kewei Zhao, Satish C. Myneni, Sergey Oleynik, Gerald H. Haug, Alfredo Martinez-Garcia, Daniel M. Sigman

**Affiliations:** ^a^Department of Geosciences, Princeton University, Princeton, NJ 08544; ^b^Department of Climate Geochemistry, Max Planck Institute for Chemistry, Mainz 55128, Germany; ^c^Smithsonian Tropical Research Institute, Balboa 0843-03092, Republic of Panama; ^d^Department of Earth and Environmental Sciences, Boston College, Chestnut Hill, MA 02467; ^e^Department of Geosciences, University of Louisiana at Monroe, Monroe, LA 71209; ^f^Department of Earth Sciences, ETH Zürich, Zürich 8092, Switzerland

**Keywords:** fossil fish otoliths, nitrogen isotopes, Late Cretaceous, marine biogeochemistry

## Abstract

The nitrogen isotopes of the organic matter preserved in fossil fish otoliths (ear stones) are a promising tool for reconstructing past environmental changes. We analyzed the ^15^N/^14^N ratio (δ^15^N) of fossil otolith-bound organic matter in Late Cretaceous fish otoliths (of *Eutawichthys maastrichtiensis*, *Eutawichthys zideki* and *Pterothrissus* sp.) from three deposits along the US east coast, with two of Campanian (83.6 to 77.9 Ma) and one Maastrichtian (72.1 to 66 Ma) age. δ^15^N and N content were insensitive to cleaning protocol and the preservation state of otolith morphological features, and N content differences among taxa were consistent across deposits, pointing to a fossil-native origin for the organic matter. All three species showed an increase in otolith-bound organic matter δ^15^N of ~4‰ from Campanian to Maastrichtian. As to its cause, the similar change in distinct genera argues against changing trophic level, and modern field data argue against the different locations of the sedimentary deposits. Rather, the lower δ^15^N in the Campanian is best interpreted as an environmental signal at the regional scale or greater, and it may be a consequence of the warmer global climate. A similar decrease has been observed in foraminifera-bound δ^15^N during warm periods of the Cenozoic, reflecting decreased water column denitrification and thus contraction of the ocean’s oxygen deficient zones (ODZs) under warm conditions. The same δ^15^N-climate correlation in Cretaceous otoliths raises the prospect of an ODZ-to-climate relationship that has been consistent over the last ~80 My, applying before and after the end-Cretaceous mass extinction and spanning changes in continental configuration.

## Significance

The geochemistry of fossils is used to reconstruct the ocean environment in Earth history. Our study shows the potential of organic matter within the biomineral matrix of fossil fish ear stones (otoliths) for this purpose. We report a change in nitrogen isotope ratios of fossil otoliths during the Late Cretaceous along the eastern North American continental shelf. We favor interpretation of the change as a global ocean signal, suggesting that the ocean’s oxygen-deficient zones expanded during the climate cooling of the Late Cretaceous. The large and coherent signal observed in this study bodes well for fossil otolith-bound δ^15^N as a window into past oceanic change, available even when open ocean sedimentary deposits are lacking.

In the Late Cretaceous, global climate underwent a transition from the warm “hothouse world” that characterized much of the Cretaceous to a cooler climate that persisted into the Early Cenozoic. As reconstructed from benthic and planktonic foraminiferal oxygen isotopes (1, 2), the Late Cretaceous cooling began in the late Campanian (~75 Ma) and extended into the early Maastrichtian (72 to 69 Ma), apparently associated with declining atmospheric CO_2_ (3, 4). The cooling was observed in deep waters (1, 2, 5), surface waters (6–8), and the inland Western Interior Seaway (9). Sea level reconstructions from the Atlantic Coastal Plain exhibit rapid sea level fluctuations of up to 50 m in the Late Cretaceous, suggestive of ice volume fluctuations (10, 11).

We know relatively little about the biogeochemical and ecosystem changes that occurred in response to the substantial global cooling of the Late Cretaceous, in the ocean or on land. Nitrogen (N) isotopes (the ^15^N/^14^N ratio, or δ^15^N) have the potential to provide such information, especially in marine settings (δ^15^N = ((^15^N/^14^N)_sample_/(^15^N/^14^N)_AIR, N_2__-1)*1000). N isotope reconstructions can provide insights into the δ^15^N “baseline”, which is controlled by global and regional ocean biogeochemical processes (12). To date, N isotope studies of the Late Cretaceous have focused on the δ^15^N of bulk sedimentary N (13, 14). The δ^15^N of bulk sediments can provide important information on the N cycle (15), but, in most sedimentary environments, the preservation of bulk sedimentary organic matter δ^15^N is questionable for recent sediments (16), let alone for millions-of-years-old Mesozoic deposits.

When fossil-producing organisms precipitate their biominerals, organic matter (OM) can be incorporated within the biomineral matrix, and this OM can be subsequently protected from diagenesis (17, 18). Recently, δ^15^N analysis of OM bound in the mineral matrix has been applied to diatoms (19–21), foraminifera (22, 23), corals (24, 25), mollusks (26, 27), tooth enamel (28, 29), and fish otoliths (30, 31). In the case of heterotrophic organisms, fossil-bound OM δ^15^N is also affected by trophic level (28, 32–34). Ground-truthing studies have demonstrated the ability of otolith-bound OM δ^15^N (δ^15^N_oto_) to record dietary and environmental information (30, 35, 36). So far, the application of δ^15^N_oto_ has been restricted to modern and recent prehistorical otoliths (30, 37, 38), with the promise of studies in deeper (i.e., geologic) time as yet unrealized.

**Fig. 1.**
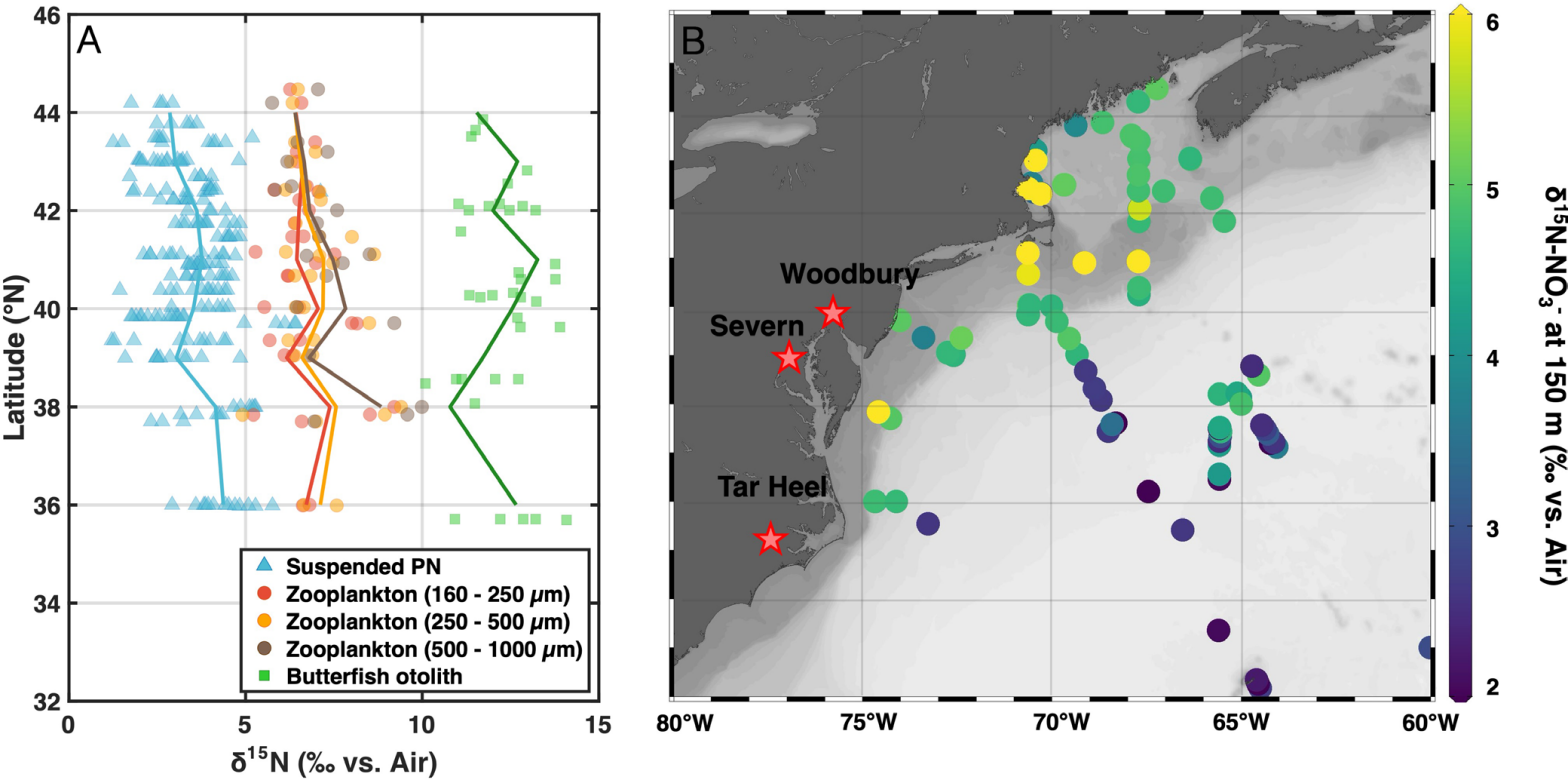
(*A*) δ^15^N measurements from the western North Atlantic continental shelf of suspended PN, zooplankton of three size ranges, and butterfish otoliths. (*B*) Map of the region, with shallow subsurface nitrate δ^15^N as colored circles (from ~150 m depth, or deepest depths along the continental margin). Stars denote the locations of three formations from which the Late Cretaceous otoliths were collected, including, from north to south, the Woodbury Formation (New Jersey, Campanian), Severn Formation (Maryland, Maastrichtian), and Tar Heel Formation (North Carolina, Campanian). The map was generated with ODV (Schlitzer, Reiner, Ocean Data View, odv.awi.de, 2023). See *SI Appendix* for background information on the samples and data in this figure.

In this study, we analyzed δ^15^N_oto_ from Late Cretaceous fossil otoliths along the Atlantic Coastal Plain of the United States east coast (Fig. 1). Fossil otoliths were recovered from sediments within the Woodbury Formation (New Jersey, USA, Campanian age), Tar Heel Formation (North Carolina, USA, Campanian age), and Severn Formation (Maryland, USA, Maastrichtian age). We selected the otoliths of *Eutawichthys maastrichtiensis*, *Eutawichthys zideki,* and *Pterothrissus* sp. These species were the three most abundant in the sedimentary deposits across the time periods of interest. The *Eutawichthys* species occur over the continental shelf at ≤200 m depth, while *Pterothrissus* sp. usually occurs at 100 to 400 m but can be found at shallower depths (39). Thus, these species should record open shelf conditions as opposed to small-scale coastal signals. We find that δ^15^N_oto_ can be measured robustly in these >66-My-old deposits. Moreover, coherent δ^15^N_oto_ changes are observed that bear on the biogeochemical cycles of the ocean over the changing climate of the Late Cretaceous.

## Results

The uncertainty in the δ^15^N_oto_ analysis is far lower than the variation among otoliths, taxa, and deposits. Replication of individual fossil otoliths yielded a δ^15^N_oto_ SD (1σ) of 0.22 ± 0.21‰, which is comparable to the long-term δ^15^N variability of an in-house coral standard (0.29‰). The blank N size for δ^15^N_oto_ analyses is between 0.24 and 0.62 nmol N. The blank contribution is generally less than 5% of total sample N (0.3 to 5.2%).

**Fig. 2.**
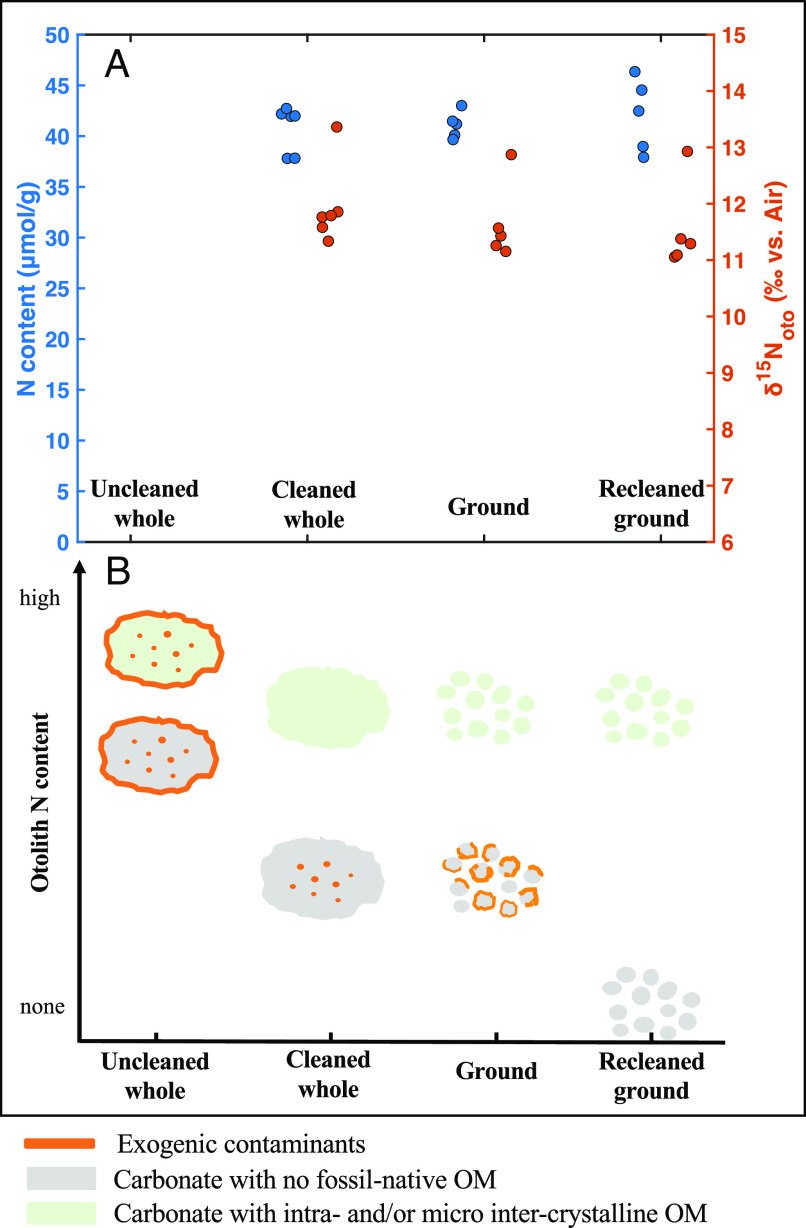
(*A*) Otolith N content (blue circles) and δ^15^N_oto_ measurements (orange circles) for well-preserved otoliths from the cleaning test. Each group represents a different cleaning treatment: (from *Left* to *Right*) uncleaned whole otoliths, cleaned whole otoliths, ground otolith powder from the cleaned whole otoliths, and ground otolith powders with a second cleaning/recleaning. The lower bound of the δ^15^N_oto_ axis range (6‰) is approximately the lowest possible δ^15^N_oto_ for a modern fish in this region, taken as the suspended PN δ^15^N (3‰) in the region plus 3‰ for the trophic level δ^15^N increase. (*B*) Expected otolith N content changes for different cleaning steps. The colors used for otolith carbonate matrix represent the scenarios without fossil-native organic matter (gray) and with intracrystalline and/or micro-intercrystalline fossil-native organic matter (green).

**Fig. 3.**
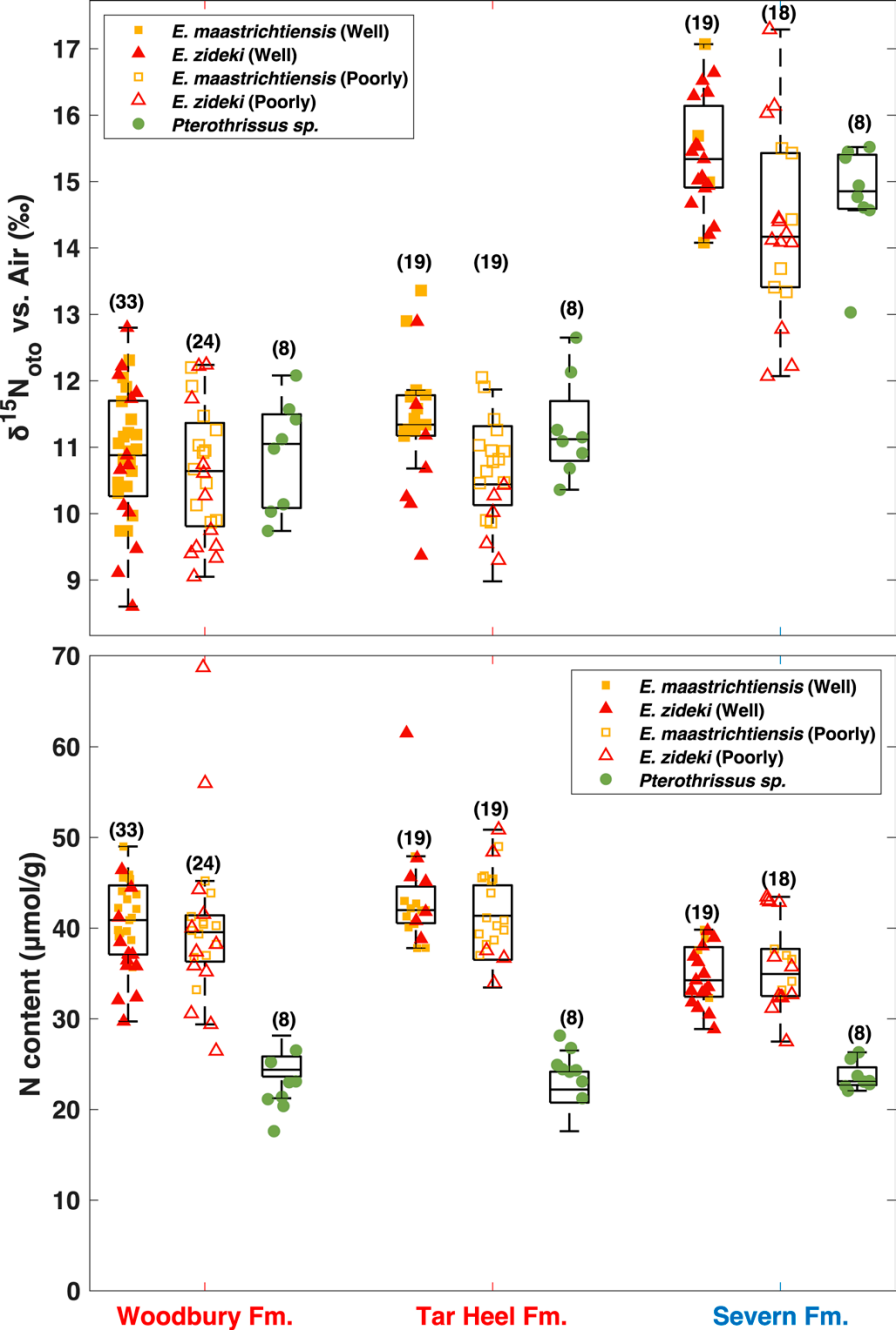
Box plots of otolith-bound δ^15^N and N content measurements for all taxa from Woodbury Formation, Tar Heel Formation and Severn Formation. *E. maastrichtiensis* results are denoted in yellow squares, *E. zideki* in red triangles. Filled symbols represent measurements from well-preserved otoliths, open symbols are measurements from poorly preserved otoliths. Green circles are for *Pterothrissus* sp., the preservation of which were not evaluated. The numbers in parentheses indicate the number of fossil otoliths analyzed.

Mineralogical identification on a subset of sample fossil otoliths, including otoliths of both *Eutawichthys* spp. and *Pterothrissus* sp., indicates that all have maintained aragonitic mineralogy (*SI Appendix*, Figs. S8 and S9). This is despite the microscopic characterization of many of the *E. zideki* otoliths as being poorly preserved (*SI Appendix*, Fig. S8). In our cleaning tests, δ^15^N_oto_ is not sensitive to whether the otoliths were ground prior to cleaning (Fig. 2) or to the reagent used for oxidative cleaning (*SI Appendix*, Table S1). The δ^15^N_oto_ values of qualitatively characterized “well” and “poorly” preserved otoliths also cover a similar range (*SI Appendix*, Fig. S4 and Table S2). Moreover, across the geologic deposits, δ^15^N_oto_ was similar between well-preserved and poorly preserved *Eutawichthys* spp. otoliths (10.9‰ vs. 10.6‰, 11.4‰ vs. 10.7‰, and 15.4‰ vs. 14.3‰ for Woodbury, Tar Heel, and Severn Formations, respectively; Fig. 3).

Otolith N content is similar between the two investigated species in the genus *Eutawichthys* (40.5 ± 4.0 μmol N/g in *E. maastrichtiensis* and 38.2 ± 7.6 μmol/g in *E. zideki*), while the otolith N content of *Pterothrissus* sp. is distinct (23.5 ± 2.4 μmol/g) (Fig. 3). For each taxon, the otolith N content is comparable across all deposits, yielding similar taxon-level differences in all three deposits (Fig. 3).

The δ^15^N_oto_ values of all species are similar to one another for the Campanian-age Woodbury Formation (New Jersey) and Tar Heel Formation (North Carolina) (Fig. 3). *Eutawichthys* spp. δ^15^N_oto_ is 10.77 ± 0.98‰ in the Woodbury Formation and 11.06 ± 1.06‰ in the Tar Heel Formation; the *Pterothrissus* sp. δ^15^N_oto_ is 10.89 ± 0.83‰ in the Woodbury Formation and 11.28 ± 0.75‰ in the Tar Heel Formation. Within a formation, the mean δ^15^N_oto_ of genera *Eutawichthys* and *Pterothrissus* are not significantly different, regardless of apparent preservation state (Student’s *t* test, *P* = 0.76 in Woodbury Formation, *P* = 0.58 in Tar Heel Formation, *P* = 0.84 in Severn Formation). Notably, however, the δ^15^N_oto_ in the Severn Formation (of Maastrichtian age) is clearly higher than that in the two Campanian formations (Fig. 3; Student’s *t* test, *P* ≪ 0.01 for mean δ^15^N_oto_ of all Maastrichtian otoliths comparing to all Campanian otoliths). This δ^15^N_oto_ increase is observed in both *Eutawichthys* spp. (10.89 ± 1.02‰ in Campanian, 14.87 ± 1.25‰ in Maastrichtian) and *Pterothrissus* sp. (11.08 ± 0.79‰ in Campanian, 14.78 ± 0.80‰ in Maastrichtian).

## Discussion

### Insights into Otolith-Bound N from N Content and Cleaning Tests.

Modern otoliths are characterized by different N contents across taxa (30, 35, 40), which is also observed in our study of fossil otoliths. The fossil otoliths of one genus in our study, *Eutawichthys*, have on average higher N content than most modern otoliths from extant species studied so far, whereas the fossil otolith N content of *Pterothrissus* sp. falls within the typical modern range (30, 35). Notably, the taxon-specific N contents of fossil otoliths, including their intertaxonomic differences, are consistent across all three deposits (Fig. 3*B*). These findings are consistent with the otolith-bound N being native to the otolith and preserved in these geologic settings.

The cleaning tests offer further insight into the fossil otolith-bound N (Fig. 2). Whether the analyzed whole otoliths were 1) cleaned, 2) cleaned and ground, or 3) cleaned, ground, and recleaned, N content was remarkably similar, as was δ^15^N_oto_ (Fig. 2*A*). If the organic matter (OM) in question was present dominantly as accumulations in the pore spaces within otoliths (orange specs in gray matrix in Fig. 2*B*), then N content should have declined upon grinding and recleaning. The lack of change in N content (and δ^15^N_oto_) with recleaning argues that the OM is intracrystalline or micro-intercrystalline, a distribution more consistent with a fossil-native origin than with diagenetic incorporation.

Additionally, the otolith N content of each taxon is independent of which cleaning reagent was used (*SI Appendix*, Fig. S3 and Table S1). In the case of using persulfate reagent for cleaning, this is the same reagent used, after dissolving the biomineral in acid, for N oxidation to nitrate in δ^15^N analysis. Therefore, the survival of this OM through the cleaning process can only be explained by physical protection by the biomineral matrix, as opposed to OM survival due to chemical recalcitrance (*SI Appendix*, Fig. S3*A* and Table S1). The fossil-bound N must be dominantly occurring within the otolith grains, again pointing to residence of the OM in the least diagenetically accessible components of the biomineral structure and, thus, a fossil-native origin.

Finally, we observe similar N content and δ^15^N_oto_ between well-preserved and poorly preserved *Eutawichthys* spp. otoliths (Fig. 3 and *SI Appendix*, Fig. S4), implying that postdepositional alteration to the organic N within these fossil otoliths was unimportant in all three deposits. The slightly lower N content could be due to loss of a small proportion of the otolith-bound organic N when it was exposed to diagenesis. However, there is no evidence of any N isotopic change (*SI Appendix*, Table S2), implying that any exposure of fossil-bound N leads to its complete loss, consistent with its dominant occurrence as peptide and/or free amino acid N (35), which should be diagenetically labile. These findings conform with cleaning experiments on other fossil types (41). All told, both the methodological tests and the results from the different deposits point to otolith-bound OM as a unique window into ancient organisms and their environments.

### Causes of Variation in δ^15^N_oto_.

**Fig. 4.**
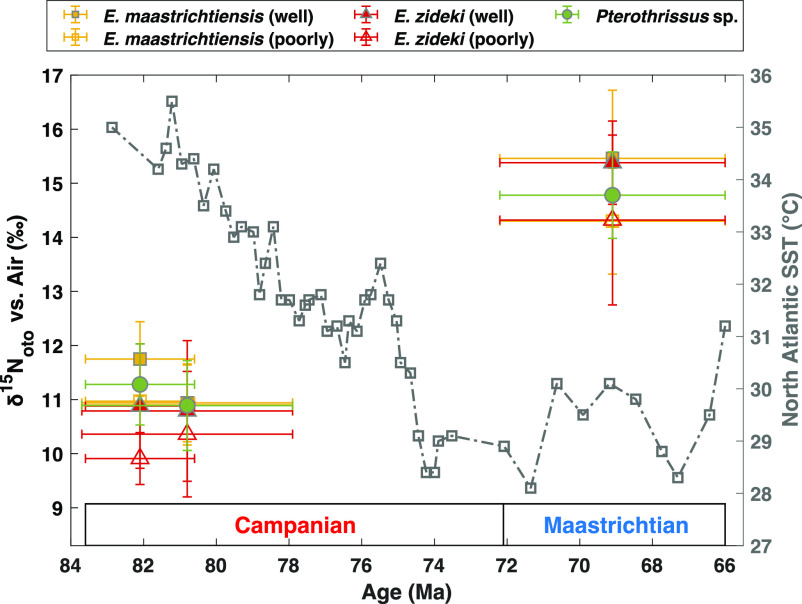
Compilation of δ^15^N_oto_ results, plotted with Late Cretaceous sea surface temperature (SST) for the western North Atlantic reconstructed with TEX86 (6). The SST trend is in gray rectangles with dotted-dash line. Colored symbols are average δ^15^N_oto_ of each taxon from each formation (see legend). Vertical error bars are calculated from the SD (1SD) of measured δ^15^N_oto_. Age constraints (plotted age values and horizontal error bars) of Campanian otoliths are based on estimates from refs. 39 and 42. The age of Maastrichtian otoliths uses the age estimates for the entire epoch (sourced from the International Chronostratigraphic Chart).

Within a formation, the intrataxon δ^15^N_oto_ SD are between 0.75 and 1.25‰ (Fig. 3). This variability is not clearly different from the variability observed from the modern butterfish otoliths (0.99‰; Fig. 1), even though the modern otoliths are all from the same ecosystem. Considering that the fossil otoliths were collected from geologic formations that span millions of years and cover large coastal regions, the formation-level variability is remarkably small. Similar δ^15^N_oto_ variability across formations also suggests similar degrees of ecological and temporal averaging for all deposits. Regardless, the within-deposit variability in δ^15^N_oto_ is minor compared to the δ^15^N_oto_ shift of ~4‰ (Fig. 4) from the Campanian formations to the Maastrichtian formation. The δ^15^N_oto_ increase is consistent across all three species (Figs. 3 and 4). It could be attributed to a) an increase in trophic level of both *Eutawichthys* spp. and *Pterothrissus* sp. fishes (34) and/or b) an increase in “baseline” δ^15^N, that is, the δ^15^N of the photosynthetic OM available for consumption by higher trophic levels (12, 15). We consider both potential explanations below.

### Changes in Fish Trophic Level.

Fish tissue δ^15^N [which is recorded by δ^15^N_oto_; (30)] varies with trophic level, with higher trophic level fishes having a higher δ^15^N. Trophic level can vary based on prey availability and prey preferences of the fish, even within a species (43). Modern fishes of Albulidae and Berycidae, the taxonomic families of *Pterothrissus* sp. and *Eutawichthys* spp., respectively, both consume diel-migrating zooplankton, worms, and/or small fishes (44, 45). As there is no known major ecosystem turnover during the Campanian-to-Maastrichtian interval, the likelihood of fundamental dietary change in these fishes is low. Another potential driver of trophic level shift is fish size, as larger fishes can occupy higher trophic position within a given species (46, 47). However, the ranges of fish size estimated from otolith weight greatly overlap between the Campanian and Maastrichtian (*SI Appendix*, Fig. S7). Moreover, there is no significant correlation between δ^15^N_oto_ and fossil otolith weight, whether considering all otoliths or only the well-preserved otoliths (*SI Appendix*, Fig. S7).

Arguably the strongest evidence against trophic change as the cause of the Campanian-to-Maastrichtian δ^15^N_oto_ increase is the similarity of the δ^15^N_oto_ increase observed in all three taxa. If the δ^15^N_oto_ increase were due to trophic level change, both *Eutawichthys* species and the *Pterothrissus* species would need to have altered their diets concomitantly. In addition, the 4‰ amplitude of the δ^15^N_oto_ increase would correspond to an increase in one to two trophic levels, depending on the trophic discrimination factor [(48) and references therein]. Such a large increase would be extraordinary for species in separate families generating identifiably distinct otoliths, and it would also require significant restructuring of marine food webs between the Campanian and Maastrichtian. There are no obvious drivers for such a dramatic change in fish diet between studied time periods. Thus, the δ^15^N_oto_ change most likely records a Campanian-to-Maastrichtian increase in baseline δ^15^N along the continental shelf on the mid-Atlantic western margin.

### On-Shelf Baseline δ^15^N Gradients.

Along the modern mid-Atlantic western margin at the depth ranges believed to be preferred by the taxa in our study, there is little evidence for a substantial along-shore δ^15^N gradient. The δ^15^N of multiple N pools does not exhibit a latitudinal change along the continental shelf between 36°N and 44°N (Fig. 1*A*), implying little latitudinal variation in the δ^15^N of the nitrate supply to the shelf ecosystem. The offshore nitrate δ^15^N decline (Fig. 1*B*) reflects the well-documented low-δ^15^N nitrate pool of the subtropical gyre thermocline, the low density of which prevents direct exchange with the shelf (49). The sporadic elevation in nitrate δ^15^N in some water samples from the shelf (Fig. 1*B*) reflects partial assimilation of nitrate, not variation in the δ^15^N of the nitrate supply, as signaled by the lower nitrate concentrations of these samples (*SI Appendix*, Fig. S2). Near the shelf-slope break, the nitrate δ^15^N increase from south to north is 1‰ or less over the latitudinal range of our data (50). These modern observations aside, the highest Cretaceous otolith δ^15^N measurements are from Severn Formation otoliths in Maryland, located between the New Jersey and North Carolina deposits, while the otoliths from these northernmost and southernmost locations exhibit similar δ^15^N_oto_ (Fig. 3). All of these observations argue against a role for an along-shore gradient in the δ^15^N differences among the three deposits.

A small outer-shelf-to-coast increase in δ^15^N (of 1 to 2‰) is observed in particulate nitrogen (PN), zooplankton, and fish tissues on the modern Northeast coast (51–53). This may arise in part from fixed N inputs at the coast contrasting with lower δ^15^N of nitrate from the open North Atlantic (Fig. 1*B*). However, the magnitude of this δ^15^N gradient is known to be attenuated at higher trophic levels for pelagic fishes, likely due to the transport of plankton and the movement of nekton (53). The Cretaceous species investigated here are expected to be found at various shelf depths and certainly not restricted to nearshore (39, 42). Therefore, cross-shelf δ^15^N variation, in isolation, is also an unlikely explanation for the ~4‰ increase δ^15^N_oto_ from Campanian to Maastrichtian.

### Baseline δ^15^N Change at the Regional or Global Scale.

Studies of fossil-bound N isotopes have found evidence of major shifts in baseline δ^15^N through the Cenozoic, on time scales from millions of years (54, 55), to orbital cycles (56), to millennial timescales and shorter (57, 58). These prior findings are believed to reflect changes in the ocean N cycle on a regional and/or global basis. Moreover, as discussed above, we find little evidence for a strong along-shelf or outer-shelf-to-coast baseline δ^15^N gradient across the latitudes of interest (Fig. 1). Thus, the δ^15^N_oto_ elevation of ~4‰ observed in the Maastrichtian relative to the Campanian likely reflects a baseline δ^15^N change on a scale greater than that of along- and cross-shelf gradients.

Baseline δ^15^N is directly related to the δ^15^N of the shallow subsurface nitrate that is mixed or upwelled into the euphotic zone (22–24, 59). On the western margin of the North Atlantic, the tilted pycnocline exposes nitrate from below the thermocline that underlies the subtropical gyre (49). This subthermocline nitrate is ~4.8‰, similar to the δ^15^N of deep ocean nitrate (60). In the open ocean, the center of the gyre is characterized by thermocline nitrate with a lower δ^15^N (~3‰), due to the remineralization of newly fixed N transported from the south (61). The δ^15^N of the regional nitrate supply to the continental shelf ecosystem could be affected by the strength of N_2_ fixation in the North Atlantic and/or changes in the exchange between the gyre thermocline and the shelf, such as through Gulf Stream changes.

N_2_ fixation appears to be sensitive to temperature, tending to increase at higher temperature (62). Thus, one might argue that the lower δ^15^N_oto_ of the Campanian was due to the higher temperature of the Campanian causing an increase in N_2_ fixation. However, on a global basis and over time scales of thousands of years and longer, N_2_ fixation must balance ocean N loss, which dominantly occurs by denitrification (63). Evidence from both the modern ocean and paleoceanographic studies indicate that N_2_ fixation responds to N loss rates, through the N-depleted, P-bearing surface waters produced by N loss (61, 63). While temperature changes may alter the surface ocean distribution of N_2_ fixation, we know of no reason that the Campanian-to-Maastrichtian cooling would have shifted N_2_ fixation from the North Atlantic to other regions.

The nitrate supply δ^15^N might change due to changes in regional circulation. For example, a weaker margin-to-gyre surface density gradient may have increased the input of the lower-δ^15^N nitrate of the subtropical gyre thermocline to the margin surface waters (64). However, given modern isotopic gradients in the region, the maximum δ^15^N variation due to such a change is ~2.5‰ (49). Thus, even a total lack of margin-to-gyre density gradient during the warmer Campanian transitioning to the modern situation during the Maastrichtian would be insufficient to explain the 4‰ increase in δ^15^N_oto_.

Last, changes in the sedimentary denitrification on the continental shelf can be driven by the sea level change. Eustatic sea level reconstructions in the Late Cretaceous suggest fluctuations of up to 40 m (10, 11), with the possibility that reduced continental shelf area decreased sedimentary denitrification during the colder Maastrichtian. Sedimentary denitrification usually does not itself express significant isotopic fractionation (65). Rather, the dominant regional isotopic signal appears to be from compensatory N_2_ fixation: Sedimentary denitrification on the shelf tends to stimulate N_2_ fixation on a regional basis (57). A reduction in shelf sedimentary denitrification associated with cooling-induced ice growth would tend to reduce regional N_2_ fixation, which would decrease its tendency to lower thermocline nitrate δ^15^N in the western North Atlantic. However, even for the large sea level drop of ~125 m of the late Pleistocene glacial maxima, foraminifera-bound δ^15^N (FB-δ^15^N) increased by only ~3‰ in the South China Sea (57). Thus, the possible Late Cretaceous sea level decline of 40 m would likely be insufficient to explain the observed 4‰ Campanian-to-Maastrichtian rise in δ^15^N_oto_.

Given the shortcomings of the regional mechanisms described above, we progress to the interpretation that the Campanian-to-Maastrichtian δ^15^N_oto_ increase was due to a baseline δ^15^N increase on the ocean basin and/or global ocean scale. The subsurface nitrate δ^15^N of the whole ocean is sensitive to the proportion of denitrification in the water column versus the sediments (66), with a range of secondary influences (67, 68). In this context, the δ^15^N_oto_ increase implies a decrease in sedimentary denitrification and/or an increase in water column denitrification. With the large (~120 m) sea level drop during late Pleistocene glacial maxima, there is no sign that the global mean ocean nitrate δ^15^N rose by anything comparable to the 4‰ increase observed in δ^15^N_oto_ from the Campanian to the Maastrichtian (67). Thus, we tend to discount a global decline in sedimentary denitrification as the cause of the δ^15^N_oto_ increase.

The alternative global ocean explanation for the Campanian-to-Maastrichtian δ^15^N_oto_ rise is an increase in water column denitrification, which likely requires expansion of the ocean’s suboxic zones. The average oxygen content of ocean water is expected to be lower under warmer climates (69). However, recent model studies suggest that, even as warming causes the global ocean to lose oxygen on average, the thermocline-hosted oxygen-deficient zones (ODZs) can shrink (70, 71). The latter change would reduce water column denitrification, lowering nitrate δ^15^N most near the ODZs (today, sited in the eastern tropical Pacific and the Arabian Sea) but also throughout the global pycnocline (68). FB-δ^15^N data from multiple warm periods in the Cenozoic—the Paleogene-Eocene Thermal Maximum, Early Eocene Climate Optimum and Middle Miocene Climate Optimum—are consistent with smaller ODZs under warmer climate. In those warm periods, FB-δ^15^N was substantially lower, with the largest δ^15^N declines occurring in the tropical Pacific, closest to the eastern tropical Pacific ODZs (54, 55, 72), pointing to ODZ shrinkage (73). While this change would have most strongly reduced nitrate δ^15^N near the shrinking ODZ, it also would have engendered a global ocean nitrate δ^15^N decline because of a decrease in the global ocean’s ratio of water column to sedimentary denitrification. Thus, the Campanian-to-Maastrichtian δ^15^N_oto_ increase can be explained by the same climate sensitivity of ODZs, with ODZs expanding and water column denitrification accelerating as climate cooled into the Maastrichtian.

## Conclusions

From a study of Late Cretaceous deposits along North America’s eastern margin, we find that paleoenvironmental signals are preserved in the δ^15^N of fossil fish otoliths as old as ~80 Ma. This opens a new avenue by which to reconstruct paleoenvironmental and paleoecological conditions in Cenozoic and pre-Cenozoic Earth history. For their sustenance, fish ultimately rely on regional phytoplankton production, which records the isotopic composition of fixed N in the environment; thus, fish otoliths provide a potential probe of past changes in the ocean’s N cycle. Further, otoliths are diagnostic of specific taxa and can be used to reconstruct the trophic levels of those taxa and potentially the trophic structure of the food web in which they live. In addition, fish otoliths have the potential to fill a gap between fossils from organisms occupying low trophic levels (e.g., planktonic foraminifera and the shells of bivalves and mollusks) and top predators (e.g., shark teeth), all of which can be measured for their fossil-bound N isotopic composition (22, 24, 26, 28, 29). Studies of the ocean during the pre-Cenozoic are hampered by the subduction-driven loss of open ocean seafloor over time and the limited evolutionary history of planktonic foraminifera. As fish have a longer evolutionary history and otoliths accumulate in ocean margin sediments, they may prove particularly useful for studies of pre-Cenozoic and even pre-Mesozoic Earth history.

For all three fish taxa analyzed in this study, a δ^15^N_oto_ increase of ~4‰ was observed from the Campanian to the Maastrichtian, a change that we interpret to reflect not trophic level change but rather a baseline δ^15^N increase at the regional and/or global scale. There are several plausible explanations for this baseline δ^15^N change. We favor a change in the nitrate δ^15^N supplied to the North American northeast continental shelf system. This δ^15^N_oto_ change is consistent with the negative correlation between global temperature and ocean oxygen-deficient zone extent that has been reconstructed from foraminifera-bound N isotopes and other proxies from the Cenozoic (54, 55, 72, 73). The observed δ^15^N_oto_ increase may reflect that water column denitrification increased from the warmer Campanian to the cooler Maastrichtian. If so, the results indicate that linkages between global climate and ocean oxygen-deficient zone extent were similar between the Mesozoic and Cenozoic Eras, despite the end-Cretaceous mass extinction (74) and changes in ocean basin geometry from the Cretaceous to the Cenozoic (75), suggesting that this biogeochemically and ecologically important sensitivity to global climate is a permanent feature of Earth’s ocean.

## Materials and Methods

All otoliths were sagittal otoliths, typically the largest of the three paired otoliths in the teleost fishes and the most frequently examined in paleontological and geochemical studies (76). Pollen studies, nannofossils, and foraminiferal biozonation of the Woodbury Formation place it in the early-middle Campanian, with an age of 83.6 to 77.9 Ma (39). The Tar Heel Formation is also dated to early-middle Campanian, based on nannofossils biozones and Sr isotopes (77), and it has similar sedimentologic and stratigraphic features to the Woodbury Formation (42). The Severn Formation represents the Atlantic coastal environment in the Maastrichtian (78). Collected from these formations are otoliths of the three most abundant taxa: *E. maastrichtiensis*, *E. zideki,* and *Pterothrissus* sp. All have been interpreted to inhabit the Cretaceous the upper to middle continental shelf (39, 78). We know little about the diet of *Eutawichthys* spp., as they went extinct at the Cretaceous/Paleogene boundary and do not have typical modern representatives. The closely related modern representative of *Pterothrissus* sp. is *Nemoossis belloci*, an endemic species in the eastern Atlantic Ocean (79), is inferred as a nektonic shelf inhabitant that feeds on zooplankton and polychaetes.

### Otolith Preservation.

The preservation condition of fossil otoliths in our study was evaluated by the presence or absence of salient morphological features. These features are located primarily on the inner face of the sagitta, although some may deal with the entire specimen. Morphological features employed to determine the preservation are listed in *SI Appendix*, Tables S3 and S4. Specimens with four features or less are designated as poorly preserved, whereas those with six or more are denoted as well preserved. If majorities of the morphological features are present, even if an otolith shows eroded margins and appear to be partially eroded, it could still be deemed as well preserved. Characteristics such as coloration and staining do not affect the evaluated preservation unless they obliterate the morphological features. The morphological features are specific for the otoliths used in this study, namely *E. maastrichtiensis* and *E. zideki*. Preservation categories for *Pterothrissus* sp. were not determined in this study.

### Otolith-Bound δ^15^N Nitrogen Isotopic Analysis.

All the otoliths were imaged twice under reflective light (Leica KL1500 LCD) with a camera attached to a stereomicroscope (Leica S6D), before and after they underwent an initial cleaning as individual whole otoliths. Each otolith was first cleaned with 10 mL 2% sodium polyphosphate solution under ultrasonication for 10 s. To remove any metal oxide coatings, 10 mL sodium dithionite solution (31 g sodium citrate + 10 g sodium bicarbonate + 25 g sodium hydrosulfite in 500 mL deionized water) was aliquoted into the sample vials, which were placed in an 80 °C water bath for an hour. Finally, to remove external organic contaminants, 10 mL of “bleach” solution (sodium hypochlorite, 10 to 15% available chlorine; Sigma-Aldrich reagent grade) was aliquoted into the sample vials, which were shaken overnight (~15 h). Samples were rinsed with deionized water three times after each cleaning step and were dried at 55 °C. Powdered otolith samples were portioned from the ground otoliths and cleaned again with bleach (or a persulfate reagent in the case of cleaning tests).

The “persulfate-denitrifier” (or “oxidation-denitrifier”) method was used for fossil-bound organic matter δ^15^N analysis (19, 22, 24, 30). For each δ^15^N measurement, 2 to 5 mg cleaned otolith powder was weighed into precombusted 4 mL vials. Additionally, 2 to 3 subsamples of in-house coral standard CBS2019 (*Diploria labyrinthiformis*, 63 to 250 μm grain size) and otolith standard HDS (*Melanogrammus aeglefinus*, 250 to 425 μm grain size) were analyzed with each sample batch. Each sample or standard was dissolved in 80 μL 4 mol/L HCl to remove the mineral matrix, and 1 mL aliquots of a persulfate-based oxidizing reagent (produced by dissolving 1 g recrystallized potassium persulfate and 2 g sodium hydroxide in 100 mL high-purity deionized water) were then added to each vial to oxidize the organic nitrogen to nitrate (NO_3_^−^). Samples were autoclaved under 121 °C for 1 h. After oxidation, all samples were *p*H-adjusted to 5 to 7, and NO_3_^−^ concentration was measured by conversion to nitric oxide in acidic vanadium solution followed by chemiluminescence detection (80) with a chemiluminescence NO_x_ analyzer (Model 200E, Teledyne Instruments). Samples were then injected into vials containing concentrated culture of a strain of *Pseudomonas chlororaphis* that lacks nitrous oxide reductase activity to quantitatively convert the NO_3_^−^ to nitrous oxide (N_2_O) (81, 82). The δ^15^N of the resulting N_2_O was measured with a custom-built in-line preparation and purification system connected to a gas-source isotope ratio mass spectrometer (GC-IRMS, Thermo Fisher MAT253) (82, 83). δ^15^N measurement was corrected for the N blank associated with the persulfate oxidation protocol (*SI Appendix*). Isotopic ratios of the measured samples are reported relative to the international reference, N_2_ in air.

### Fossil Otolith Mineralogy Determination.

A Fourier transform infrared spectrometer (Vertex 80v, Bruker Corporation) was used to differentiate calcite and aragonite for the fossil otoliths. The IR spectroscopy uses a mercury–cadmium–telluride detector and measures attenuated total reflection from the sample on a diamond crystal. Powdered sample was placed on the diamond crystal and was compressed to ensure full coverage of the crystal surface with sample materials. Each sample was measured every 2 cm^−1^ between 600 and 3,998 cm^−1^ wavenumber and was scanned 500 times to acquire the attenuated reflection. The FTIR spectra were corrected by normalizing the absorbance in reference to the maximum absorbance after subtracting the regression baseline modeled from the 1,800 to 3,998 cm^−1^.

## Supplementary Material

Appendix 01 (PDF)

Dataset S01 (XLSX)

Dataset S02 (XLSX)

## Data Availability

All study data are included in the article and/or supporting information.

## References

[1] O. Friedrich, R. D. Norris, J. Erbacher, Evolution of middle to Late Cretaceous oceans—A 55 m.y. record of Earth’s temperature and carbon cycle. Geology **40**, 107–110 (2012).

[2] B. T. Huber, K. G. MacLeod, D. K. Watkins, M. F. Coffin, The rise and fall of the Cretaceous Hot Greenhouse climate. Global Planet. Change **167**, 1–23 (2018).

[3] S. K. Hong, Y. I. Lee, Evaluation of atmospheric carbon dioxide concentrations during the Cretaceous. Earth Planet. Sci. Lett. **327–328**, 23–28 (2012).

[4] D. L. Royer, CO2-forced climate thresholds during the Phanerozoic. Geochim. Cosmochim. Acta **70**, 5665–5675 (2006).

[5] B. S. Cramer, K. G. Miller, P. J. Barrett, J. D. Wright, Late Cretaceous-Neogene trends in deep ocean temperature and continental ice volume: Reconciling records of benthic foraminiferal geochemistry ( δ 18 O and Mg/Ca) with sea level history. J. Geophys. Res. **116**, C12023 (2011).

[6] C. Linnert , Evidence for global cooling in the Late Cretaceous. Nat. Commun. **5**, 4194 (2014). 24937202 10.1038/ncomms5194 PMC4082635

[7] C. L. O’Brien , Cretaceous sea-surface temperature evolution: Constraints from TEX86 and planktonic foraminiferal oxygen isotopes. Earth Sci. Rev. **172**, 224–247 (2017).

[8] N. Thibault, R. Harlou, N. H. Schovsbo, L. Stemmerik, F. Surlyk, Late Cretaceous (Late Campanian–Maastrichtian) sea surface temperature record of the Boreal Chalk Sea. Clim. Past **12**, 5049–5071 (2015), 10.5194/cpd-11-5049-2015.

[9] K. J. Dennis, J. K. Cochran, N. H. Landman, D. P. Schrag, The climate of the Late Cretaceous: New insights from the application of the carbonate clumped isotope thermometer to Western Interior Seaway macrofossil. Earth Planet. Sci. Lett. **362**, 51–65 (2013).

[10] M. A. Kominz , Late Cretaceous to Miocene sea-level estimates from the New Jersey and Delaware coastal plain coreholes: An error analysis. Basin Res. **20**, 211–226 (2008).

[11] K. G. Miller , Late Cretaceous chronology of large, rapid sea-level changes: Glacioeustasy during the greenhouse world. Geology **31**, 585 (2003).

[12] D. M. Sigman, F. Fripiat, “Nitrogen isotopes in the ocean” in Encyclopedia of Ocean Sciences, J. K. Cochran, H. J. Bokuniewicz, P. L. Yager, Eds. (Academic Press, ed. 3, 2019), pp. 263–278.

[13] C. K. Junium, S. R. Meyers, M. A. Arthur, Nitrogen cycle dynamics in the Late Cretaceous Greenhouse. Earth Planet. Sci. Lett. **481**, 404–411 (2018).

[14] J. Sepúlveda , Stable isotope constraints on marine productivity across the Cretaceous-Paleogene mass extinction. Paleoceanogr. Paleoclimatol. **34**, 1195–1217 (2019).

[15] G. H. Rau, M. A. Arthur, W. E. Dean, 15N/14N variations in Cretaceous Atlantic sedimentary sequences: Implication for past changes in marine nitrogen biogeochemistry. Earth Planet. Sci. Lett. **82**, 269–279 (1987).

[16] J. Möbius, N. Lahajnar, K.-C. Emeis, Diagenetic control of nitrogen isotope ratios in Holocene sapropels and recent sediments from the Eastern Mediterranean Sea. Biogeosciences **7**, 3901–3914 (2010).

[17] K. King, P. E. Hare, Amino acid composition of the test as a taxonomic character for living and fossil planktonic foraminifera. Micropaleontology **18**, 285 (1972).

[18] G. A. Sykes, M. J. Collins, D. I. Walton, The significance of a geochemically isolated intracrystalline organic fraction within biominerals. Org. Geochem. **23**, 1059–1065 (1995).

[19] R. S. Robinson, B. G. Brunelle, D. M. Sigman, Revisiting nutrient utilization in the glacial Antarctic: Evidence from a new method for diatom-bound N isotopic analysis. Paleoceanography **19**, PA3001 (2004).

[20] A. Shemesh, S. A. Macko, C. D. Charles, G. H. Rau, Isotopic evidence for reduced productivity in the glacial Southern Ocean. Science **262**, 407–410 (1993). 17789948 10.1126/science.262.5132.407

[21] D. M. Sigman, M. A. Altabet, R. Francois, D. C. McCorkle, J. Gaillard, The isotopic composition of diatom-bound nitrogen in Southern Ocean sediments. Paleoceanography **14**, 118–134 (1999).

[22] H. Ren, D. M. Sigman, R. C. Thunell, M. G. Prokopenko, Nitrogen isotopic composition of planktonic foraminifera from the modern ocean and recent sediments. Limnol. Oceanogr. **57**, 1011–1024 (2012).

[23] S. M. Smart , Ground-truthing the planktic foraminifer-bound nitrogen isotope paleo-proxy in the Sargasso Sea. Geochim. Cosmochim. Acta **235**, 463–482 (2018).

[24] X. T. Wang , Isotopic composition of carbonate-bound organic nitrogen in deep-sea scleractinian corals: A new window into past biogeochemical change. Earth Planet. Sci. Lett. **400**, 243–250 (2014).

[25] X. T. Wang , Isotopic composition of skeleton-bound organic nitrogen in reef-building symbiotic corals: A new method and proxy evaluation at Bermuda. Geochim. Cosmochim. Acta **148**, 179–190 (2015).

[26] S. Das, E. J. Judd, B. T. Uveges, L. C. Ivany, C. K. Junium, Variation in δ15N from shell-associated organic matter in bivalves: Implications for studies of modern and fossil ecosystems. Palaeogeogr. Palaeoclimatol. Palaeoecol. **562**, 110076 (2021).

[27] D. P. Gillikin , High-resolution nitrogen stable isotope sclerochronology of bivalve shell carbonate-bound organics. Geochim. Cosmochim. Acta **200**, 55–66 (2017).

[28] E. R. Kast , Cenozoic megatooth sharks occupied extremely high trophic positions. Sci. Adv. **8**, eabl6529 (2022). 35731884 10.1126/sciadv.abl6529 PMC9217088

[29] J. N. Leichliter , Nitrogen isotopes in tooth enamel record diet and trophic level enrichment: Results from a controlled feeding experiment. Chem. Geol. **563**, 120047 (2021).

[30] J. A. Lueders-Dumont, X. T. Wang, O. P. Jensen, D. M. Sigman, B. B. Ward, Nitrogen isotopic analysis of carbonate-bound organic matter in modern and fossil fish otoliths. Geochim. Cosmochim. Acta **224**, 200–222 (2018).

[31] J. A. Lueders-Dumont , Controls on the nitrogen isotopic composition of fish otolith organic matter: Lessons from a controlled diet switch experiment. Geochim. Cosmochim. Acta **316**, 69–86 (2022).

[32] C. M. Comans , Enameloid-bound δ15N reveals large trophic separation among Late Cretaceous sharks in the northern Gulf of Mexico. Geobiology **22**, e12585 (2024). 38385603 10.1111/gbi.12585

[33] M. J. Deniro, S. Epstein, Influence of diet on the distribution of nitrogen isotopes in animals. Geochim. Cosmochim. Acta **45**, 341–351 (1981).

[34] M. Minagawa, E. Wada, Stepwise enrichment of 15N along food chains: Further evidence and the relation between δ15N and animal age. Geochim. Cosmochim. Acta **48**, 1135–1140 (1984).

[35] J. A. Lueders-Dumont , Comparison of the isotopic composition of fish otolith-bound organic N with host tissue. Can. J. Fish. Aquat. Sci. **77**, 264–275 (2020).

[36] J. M. Vandermyde, G. W. Whitledge, Otolith δ15N distinguishes fish from forested and agricultural streams in Southern Illinois. J. Freshwater Ecol. **23**, 333–336 (2008).

[37] K. Rowell, D. L. Dettman, R. Dietz, Nitrogen isotopes in otoliths reconstruct ancient trophic position. Environ. Biol. Fish **89**, 415–425 (2010).

[38] K. Vane, N. Wallsgrove, W. Ekau, B. Popp, Reconstructing lifetime nitrogen baselines and trophic position of Cynoscion acoupa from δ15N values of amino acids in otoliths. Mar. Ecol. Prog. Ser. **597**, 1–11 (2018).

[39] G. L. Stringer, L. D. Oman, R. F. Badger, Woodbury formation (Campanian) in New Jersey yields largest known Cretaceous otolith assemblage of teleostean fishes in North America. Proc. Acad. Nat. Sci. Philadelphia **165**, 15–36 (2016).

[40] E. T. Degens, Molecular structure and composition of fish otoliths. Marine Biol. **2**, 105–133 (1969).

[41] A. Martínez-García , Laboratory assessment of the impact of chemical oxidation, mineral dissolution, and heating on the nitrogen isotopic composition of fossil-bound organic matter. Geochem. Geophys. Geosyst. **23**, e2022GC010396 (2022).

[42] G. L. Stringer, D. Clements, E. Sadorf, K. Shannon, First description and significance of Cretaceous teleostean otoliths (Tar Heel Formation, Campanian) from North Carolina. East. Paleontol. **1**, 1–22 (2018).

[43] M. J. Vander Zanden, J. B. Rasmussen, Primary consumer δ13C and δ15N and the trophic position of aquatic consumers. Ecology **80**, 1395–1404 (1999).

[44] R. E. Crabtree, C. Stevens, D. Snodgrass, F. J. Stengard, Feeding habits of bonefish, Albula vulpes, from the waters of the Florida Keys. Fish. Bull. **96**, 754–766 (1998).

[45] E. Rosecchi, D. M. Tracey, W. R. Webber, Diet of orange roughy, Hoplostethus atlanticus (Pisces: Trachichthyidae) on the Challenger Plateau. New Zealand Mar. Biol. **99**, 293–306 (1988).

[46] A. Olson, A. Frid, J. Dos Santos, F. Juanes, Trophic position scales positively with body size within but not among four species of rocky reef predators. Mar. Ecol. Prog. Ser. **640**, 189–200 (2020).

[47] T. N. Romanuk, A. Hayward, J. A. Hutchings, Trophic level scales positively with body size in fishes: Trophic level and body size in fishes. Glob. Ecol. Biogeogr. **20**, 231–240 (2011).

[48] W. J. Boecklen, C. T. Yarnes, B. A. Cook, A. C. James, On the use of stable isotopes in trophic ecology. Annu. Rev. Ecol. Evol. Syst. **42**, 411–440 (2011).

[49] D. Marconi , Nitrate isotope distributions on the US GEOTRACES North Atlantic cross-basin section: Signals of polar nitrate sources and low latitude nitrogen cycling. Marine Chem. **177**, 143–156 (2015).

[50] N. Van Oostende , Variation of summer phytoplankton community composition and its relationship to nitrate and regenerated nitrogen assimilation across the North Atlantic Ocean. Deep Sea Res. Part I: Oceanogr. Res. Pap. **121**, 79–94 (2017).

[51] R. A. McKinney, A. J. Oczkowski, J. Prezioso, K. J. W. Hyde, Spatial variability of nitrogen isotope ratios of particulate material from Northwest Atlantic continental shelf waters. Estuarine Coastal Shelf Sci. **89**, 287–293 (2010).

[52] A. Oczkowski, B. Kreakie, R. A. McKinney, J. Prezioso, Patterns in stable isotope values of nitrogen and carbon in particulate matter from the Northwest Atlantic Continental Shelf, from the Gulf of Maine to Cape Hatteras. Front. Mar. Sci. **3**, 252 (2016).

[53] G. D. Sherwood, G. A. Rose, Stable isotope analysis of some representative fish and invertebrates of the Newfoundland and Labrador continental shelf food web. Estuarine Coastal Shelf Sci. **63**, 537–549 (2005).

[54] A. Auderset , Enhanced ocean oxygenation during Cenozoic warm periods. Nature **609**, 77–82 (2022). 36045236 10.1038/s41586-022-05017-0 PMC9433325

[55] E. R. Kast , Nitrogen isotope evidence for expanded ocean suboxia in the early Cenozoic. Science **364**, 386–389 (2019). 31023923 10.1126/science.aau5784

[56] B. G. Brunelle , Evidence from diatom-bound nitrogen isotopes for subarctic Pacific stratification during the last ice age and a link to North Pacific denitrification changes. Paleoceanography **22**, PA1215 (2007).

[57] H. Ren , Impact of glacial/interglacial sea level change on the ocean nitrogen cycle. Proc. Natl. Acad. Sci. U.S.A. **114**, E6759–E6766 (2017). 28760968 10.1073/pnas.1701315114 PMC5565415

[58] A. S. Studer , Ice Age-Holocene similarity of foraminifera-bound nitrogen isotope ratios in the Eastern Equatorial Pacific. Paleoceanogr. Paleoclimatol. **36**, e2020PA004063 (2021).

[59] X. T. Wang , Influence of open ocean nitrogen supply on the skeletal δ15N of modern shallow-water scleractinian corals. Earth Planet. Sci. Lett. **441**, 125–132 (2016).

[60] F. Fripiat , Nitrogen isotopic constraints on nutrient transport to the upper ocean. Nat. Geosci. **14**, 855–861 (2021).

[61] D. Marconi , Tropical dominance of N2 fixation in the North Atlantic Ocean. Global Biogeochem. Cycles **31**, 1608–1623 (2017).

[62] B. Z. Houlton, Y.-P. Wang, P. M. Vitousek, C. B. Field, A unifying framework for dinitrogen fixation in the terrestrial biosphere. Nature **454**, 327–330 (2008). 18563086 10.1038/nature07028

[63] C. Deutsch, J. L. Sarmiento, D. M. Sigman, N. Gruber, J. P. Dunne, Spatial coupling of nitrogen inputs and losses in the ocean. Nature **445**, 163–167 (2007). 17215838 10.1038/nature05392

[64] K. W. Meyer, S. V. Petersen, K. C. Lohmann, I. Z. Winkelstern, Climate of the Late Cretaceous North American Gulf and Atlantic coasts. Cretaceous Res. **89**, 160–173 (2018).

[65] M. F. Lehmann, D. M. Sigman, W. M. Berelson, Coupling the 15N/14N and 18O/16O of nitrate as a constraint on benthic nitrogen cycling. Marine Chem. **88**, 1–20 (2004).

[66] J. A. Brandes, A. H. Devol, A global marine-fixed nitrogen isotopic budget: Implications for Holocene nitrogen cycling: NITROGEN ISOTOPIC BUDGET. Global Biogeochem. Cycles **16**, 67-1–67-14 (2002).

[67] C. Deutsch, D. M. Sigman, R. C. Thunell, A. N. Meckler, G. H. Haug, Isotopic constraints on glacial/interglacial changes in the oceanic nitrogen budget. Global Biogeochem. Cycles **18**, GB4012 (2004).

[68] F. Fripiat , The impact of incomplete nutrient consumption in the Southern Ocean on global mean ocean nitrate δ15N. Global Biogeochem. Cycles **37**, e2022GB007442 (2023).

[69] N. Gruber, Warming up, turning sour, losing breath: Ocean biogeochemistry under global change. Phil. Trans. A Math. Phys. Eng. Sci. **369**, 1980–1996 (2011). 10.1098/rsta.2011.0003 21502171

[70] W. Fu, F. Primeau, J. Keith Moore, K. Lindsay, J. T. Randerson, Reversal of increasing tropical ocean hypoxia trends with sustained climate warming. Global Biogeochem. Cycles **32**, 551–564 (2018).

[71] A. Gnanadesikan, J. P. Dunne, J. John, Understanding why the volume of suboxic waters does not increase over centuries of global warming in an Earth System Model. Biogeosciences **9**, 1159–1172 (2012).

[72] S. Moretti , Oxygen rise in the tropical upper ocean during the Paleocene-Eocene Thermal Maximum. Science **383**, 727–731 (2024). 38359106 10.1126/science.adh4893

[73] A. V. Hess , A well-oxygenated eastern tropical Pacific during the warm Miocene. Nature **619**, 521–525 (2023), 10.1038/s41586-023-06104-6. 37380780

[74] L. W. Alvarez, W. Alvarez, F. Asaro, H. V. Michel, Extraterrestrial cause for the Cretaceous-tertiary extinction. Science **208**, 1095–1108 (1980). 17783054 10.1126/science.208.4448.1095

[75] Á. T. Kocsis, C. R. Scotese, Mapping paleocoastlines and continental flooding during the Phanerozoic. Earth Sci. Rev. **213**, 103463 (2021).

[76] S. Campana, Chemistry and composition of fish otoliths: Pathways, mechanisms and applications. Mar. Ecol. Prog. Ser. **188**, 263–297 (1999).

[77] W. B. Harris, J. M. Self-Trail, Late Cretaceous base level lowering in Campanian and Maastrichtian depositional sequences, Kure Beach, North Carolina. Stratigraphy **3**, 195–216 (2006).

[78] G. Stringer, W. Schwarzhans, Upper Cretaceous teleostean otoliths from the Severn Formation (Maastrichtian) of Maryland, USA, with an unusual occurrence of Siluriformes and Beryciformes and the oldest Atlantic coast Gadiformes. Cretaceous Res. **125**, 104867 (2021).

[79] K. Hidaka, Y. Tsukamoto, Y. Iwatsuki, Nemoossis, a new genus for the eastern Atlantic long-fin bonefish Pterothrissus belloci Cadenat 1937 and a redescription of P. gissu Hilgendorf 1877 from the northwestern Pacific. Ichthyol. Res. **64**, 45–53 (2017).

[80] R. S. Braman, S. A. Hendrix, Nanogram nitrite and nitrate determination in environmental and biological materials by vanadium(III) reduction with chemiluminescence detection. Anal. Chem. **61**, 2715–2718 (1989). 2619057 10.1021/ac00199a007

[81] D. M. Sigman , A bacterial method for the nitrogen isotopic analysis of nitrate in seawater and freshwater. Anal. Chem. **73**, 4145–4153 (2001). 11569803 10.1021/ac010088e

[82] M. A. Weigand, J. Foriel, B. Barnett, S. Oleynik, D. M. Sigman, Updates to instrumentation and protocols for isotopic analysis of nitrate by the denitrifier method. Rapid Commun. Mass Spectrom. **30**, 1365–1383 (2016). 27197029 10.1002/rcm.7570

[83] K. L. Casciotti, D. M. Sigman, M. G. Hastings, J. K. Böhlke, A. Hilkert, Measurement of the oxygen isotopic composition of nitrate in seawater and freshwater using the denitrifier method. Anal. Chem. **74**, 4905–4912 (2002). 12380811 10.1021/ac020113w

