# Supplementary material for: A nitrogen isotopic shift in fish otolith–bound organic matter during the Late Cretaceous
Source: Proc Natl Acad Sci U S A. 2024 Jul 29;121(32):e2322863121. doi: 10.1073/pnas.2322863121 (PMC11317583; doi:10.1073/pnas.2322863121)
Supplement: Supplementary file 1 — Appendix 01 (PDF) [file pnas.2322863121.sapp.pdf]

PNAS

Supporting Information for
A nitrogen isotopic shift in fish otolith-bound organic matter during the Late Cretaceous

Zixuan C. Rao[a,b,1], Jessica A. Lueders-Dumont[a,c,d], Gary L. Stringer[e], Yeongjun Ryu[a], Kewei Zhao[a], Satish C. Myneni[a], Sergey Oleynik[a], Gerald H. Haug[b,f], Alfredo Martinez-Garcia[b], Daniel M. Sigman[a]

[a]Department of Geosciences, Princeton University, Princeton, NJ 08544

[b]Department of Climate Geochemistry, Max Planck Institute for Chemistry, 55128 Mainz,

Germany

[c]Smithsonian Tropical Research Institute, Balboa, Republic of Panama 0843-03092

[d]Department of Earth and Environmental Sciences, Boston College, Chestnut Hill, MA 02467

[e]Department of Geosciences, University of Louisiana at Monroe, Monroe, LA 71209

[1]Corresponding author: Zixuan C. Rao

Email: zrao@princeton.edu

This PDF file includes:

- Supporting text
- Figures S1 to S9
- Tables S1 to S4
- Legends for Datasets S1 to S2
- SI References

Other supporting materials for this manuscript include the following:

- Datasets S1 to S2

Supporting Information Text

Materials and Methods

Modern oceanographic and fish otolith sample collection. The modern ocean nitrate $\delta^{15}N$ (Fig. 1B) on the continental shelf and open ocean regions are from NOAA Ecosystem Monitoring (EcoMon) program cruises (HB1502 in May 2015 and GU1608 in May 2016), CLIMODE (January-February 2006) cruises and measurements published in (1–3). Seawater sample collection for nitrate $\delta^{15}N$ was deployed at multiple depths from 3 m to 500-1000 m at depth. In the EcoMon cruises, suspended particulate nitrogen (PN) and zooplankton of various size classes are also sampled for $\delta^{15}N$ analysis. PN was collected by filtering seawater through pre-combusted 47 mm glass fiber filters. The zooplankton sampling used a 61-cm bongo sampler fitted with 165 μm nylon mesh nets. Different size fractions were separated through a series of sieves of 160 μm, 250 μm, 500 μm and 1000 μm.

The otoliths of butterfish (*Peprilus triacanthus*) were collected during the annual NOAA Northeast Fisheries Science Center (NEFSC) fall bottom trawl survey from the same geographic regions as PN and zooplankton sampling (4). The otoliths in the current study were from butterfish sampled during September-November during two time intervals (1982-1983 and 2011-2013). All fish were collected from specific NOAA strata along the U.S. northeast continental shelf (strata: 1-2, 9, 10, 14, 16, 21, 26-27, 36-37, 39-40, 42, 61-63, 70-71). Biological sampling was conducted at sea as organized by the officers, crew, and scientific team on NOAA Ship *Henry B. Bigelow*, with otolith aging conducted by the Fishery Biology Program (FBP) at NEFSC in Woods Hole, MA (4). Otoliths were

requested through the FBP sample request protocol and through in-person sampling of otolith archives with Sandy Sutherland and Eric Robillard at FBP.

The seawater nitrate $\delta^{15}N$ were analyzed using the "denitrifier" method (5) and the otoliths of the butterfish were measured with the "persulfate-denitrifier" method described in the main text. $\delta^{15}N$ of PN and zooplankton was measured with an elemental analyzer (Elementar vario MICRO cube) connected to an isotopic ratio mass spectrometer (Elemental Isoprime VisION).

Oxidative cleaning reagent test and secondary cleaning test. In previous studies using the persulfate-denitrifier method to measure the $\delta^{15}N$ of carbonate and phosphate-based fossil-bound organic N, two oxidative cleaning reagents have been used to remove external organic matter: sodium hypochlorite solution ("bleach", sodium hypochlorite with 10-15% available chlorine) and basic persulfate oxidizing reagent ("POR", 2g sodium hydroxide and 2g potassium persulfate in 100 mL deionized water). POR, when heated, has been shown to be an effective oxidant (6) and is preferred for some fossil materials (7). Also, a conceptual argument can be made for its use: The same oxidant is used to clean the biomineral as is used to oxidized the organic N that is released after dissolution of the cleaned biomineral. However, the otolith aragonite matrix of two modern taxa (pink salmon, *Oncorhynchus gorbuschai* and queen snapper, *Etelis oculatus*) have been observed to undergo recrystallization under POR cleaning, which is conducted under autoclave conditions (121°C); this led to the trapping of exogenous N within the recrystallized mineral (8). For the otoliths of the modern fishes examined, the $\delta^{15}N$ of the trapped N was similar to that of the grain-internal N (8). However, in the case of fossil otoliths, the trapped N may derive from non-native organic N with a distinct $\delta^{15}N$.

Therefore, we first performed a "reagent test" to address (a) whether bleach cleaning is adequately harsh for fossils and (b) whether POR cleaning causes recrystallization. Poorly preserved otoliths were selected for the reagent test, as they provide a more stringent test of the cleaning methods. Two otoliths of *E. maastrichtiensis* and five otoliths of *E. zideki* from the Severn Formation were separately ground and homogenized into one large sample before being portioned into six subsamples, three of which were cleaned with bleach and three with POR (Fig. S3A).

An additional test was performed to address whether otolith preservation state affects the N content and $\delta^{15}N$ (Fig. S3B). This test also investigates whether sample form (whole *vs*. powdered otoliths) influences the results. These questions are of practical importance and may also speak to the spatial distribution of the otolith-bound organic matter. In this experiment, *E. maastrichtiensis* otoliths from the Tar Heel Formation (North Carolina, USA, Campanian) were used. Well preserved and poorly preserved were each separated into two groups to be processed as whole and powdered otoliths. A subset of otoliths was each cleaned with bleach for the second time ("secondary cleaning") before $\delta^{15}N$ analysis, among which there were three well preserved and three poorly preserved otoliths respectively. The other whole otoliths (three well preserved and two poorly preserved otoliths) were analyzed directly for $\delta^{15}N$. Five well preserved and five poorly preserved otoliths were selected to be processed in powdered form. Each otolith was homogenized with a mortar and pestle before dividing the powders into two subsamples per otolith – one subsample analyzed without a secondary cleaning, and the other subsample subjected to secondary cleaning prior to $\delta^{15}N$ analysis. We selected *E. maastrichtiensis* otoliths of similar weight respectively for the test. Controlling for otolith

size in weight, which is correlated with fish length and weight within a species (9, 10), reduces the likelihood of $\delta^{15}N$ differences arising from trophic level, as larger fish can reside at higher trophic positions. Controlling for otolith length also reduces potential $\delta^{15}N$ differences arising from having larger or smaller surface-area-to-volume ratios, as surface areas might affect the degree to which diagenetic processes could affect otolith chemistry and preservation.

Blank correction. Blank N size was quantified directly by combing four oxidation blanks into one denitrifier vial and measuring it on the IRMS. Subsequently, the blank $\delta^{15}N$ is averaged from direct measurements of oxidation blanks and several means of estimation, including extrapolations of paired amino acid reference measurements from USGS40 (L-glutamic acid, $\delta^{15}N$ = -4.70‰) and USGS65 (glycine, $\delta^{15}N$ = 20.58‰), and linear extrapolation between measured $\delta^{15}N$ of amino acid reference and varying concentrations. Each sample is corrected using the size and estimated $\delta^{15}N$ of the blank with the following equations:

$$f_{sample} + f_{blank} = 1$$

$$f_{sample}\,\delta^{15}N_{sample} + f_{blank}\delta^{15}N_{blank} = \delta^{15}N_{measured}$$

where f_{blank} and f_{sample} refer to the ratio of N quantity for blank and sample, respectively.

Results and Discussion

Observed variability for N isotopic analysis. Replication of individual fossil otoliths across batches yielded a smaller $\delta^{15}N_{oto}$ standard deviation (0.22 ± 0.21‰) than the long-term $\delta^{15}N$ variability of our in-house otolith standard (0.77‰). The in-house otolith standard is homogenized from a few powdered otoliths of haddock (*Melanogrammus*

aeglefinus), sieved to 250-425 μm grain size. The haddock is known to record the ontogenetic shifts with isotopic proxies (11), and their seasonal migration (12) can also contribute to the variation of diet recorded in the fish otoliths. The otolith standard $\delta^{15}N$ variability is likely due to the heterogeneity of the $\delta^{15}N_{oto}$, as we only sample a small amount (~1-3 mg) for each $\delta^{15}N$ measurement. The "persulfate-denitrifier" method for $\delta^{15}N$ analysis is not considered as the major source for the otolith standard $\delta^{15}N$ variability, because our in-house coral standard analyzed along with each batch of fossil otoliths produces a smaller long-term $\delta^{15}N$ variability of 0.29‰. Comparable $\delta^{15}N$ variability in fossil otolith with that in our coral standard suggests the robustness of our $\delta^{15}N_{oto}$ analysis. Further, such small variability in fossil otoliths implied that the otoliths of fish taxa studied in this work do not present strong heterogeneity isotopically.

Modern N isotope patterns along the U.S. east coast. Nitrate $\delta^{15}N$ is as low as 2‰ in the shallow thermocline of the North Atlantic subtropical gyre (13), but the denser water outcrops on the western edge of the gyre along on the continental margin, yielding a $\delta^{15}N$ for the shallow subsurface nitrate supply that falls in a relatively narrow range, between 4 and 5‰ ((1); Fig. S2) and is similar along the latitudes in our study region (Fig. 1B). The uniform offshore shallow subsurface nitrate $\delta^{15}N$ implies a consistent source $\delta^{15}N$ to the shelf ecosystem recorded by our fossil otoliths. On the shelf, elevated nitrate $\delta^{15}N$ is observed sporadically (Fig. 1B), possibly due to N inputs from land or from shelf sediments but more likely the result of partial nitrate assimilation in surface waters. Indeed, we observed elevated nitrate $\delta^{15}N$ is associated with lower nitrate concentration at the sites (Fig. S2).

On the U.S. continental shelf, distinct $\delta^{15}N$ of suspended PN, zooplankton and butterfish otoliths reflect clear trophic structure in the shelf ecosystem (Fig. 1A). There is a slight decrease in the suspended PN $\delta^{15}N$ towards the higher latitude (Fig. 1A) and cases of higher PN $\delta^{15}N$ inshore (SI Appendix, Dataset S2). However, any such trends are weaker in $\delta^{15}N$ of zooplankton and are further muted in the $\delta^{15}N$ of butterfish otoliths (Fig. 1A). This is likely due to spatial averaging at higher trophic levels. Zooplankton are mixed laterally by the circulation, and nekton such as fish can move actively, both of which will cause a progressively broader spatial averaging. The fish that produced the fossil otoliths of this study appear to have been deep shelf species (14, 15), decreasing the potential for coastal N fluxes to have affected the $\delta^{15}N$ of their tissues and otoliths.

Oxidative cleaning reagent selection for fossil otoliths. The goal of the cleaning reagent test (Fig. S3A) was to assess whether the relatively harsher conditions of POR cleaning generated different results from bleach cleaning. POR has been shown to be effective for many fossil type (7, 16). However, otoliths are composed of aragonite and POR cleaning has been known to drive recrystallization in otolith aragonite of two modern fish species due to the high temperature condition (17) during the POR cleaning. Recrystallization is problematic if exogenous organics became trapped in the freshly formed recrystallized mineral instead of their removal by the oxidant. Therefore, if otoliths undergo recrystallization under POR cleaning, the otolith N content would possibly be higher, while the $\delta^{15}N_{oto}$ likely differ from that with bleach cleaning.

In our reagent test, $\delta^{15}N_{oto}$ in *E. maastrichtiensis* is 13.83 ± 0.14‰ and 13.51± 0.14‰ for bleach and POR cleaning, respectively (Table S1). This difference was not statistically significant (Student's t-test, $p = 0.0532$; Table S1). There is also no significant

difference in $\delta^{15}N_{oto}$ of the second species, *E. zideki*, for bleach and POR cleaning (15.54 ± 0.27‰ vs. 15.32 ± 0.10‰, respectively; Student's t-test, $p = 0.25$; Table S1). Neither is there a significant difference in N content between bleach and POR cleaning (Student's t-test, $p = 0.76$ for *E. maastrichtiensis* and $p = 0.33$ for *E. zideki*, Table S1). Thus, for the tested species in our study, the POR does not appear to induce recrystallization during the cleaning procedure. Also, our test indicates equal effectiveness of bleach as POR in removing exogenous contaminants. Given the potential of POR treatment to cause recrystallization of some otolith types (8), going forward, we use bleach for oxidative cleaning and recommend so for future studies on fossil otolith $\delta^{15}N_{oto}$ application.

Fossil otolith cleaning protocol and the effect of preservation. It is tempting to assume that otolith cleaning could be limited to cleaning of its external surface, where the otolith samples come in contact with the cleaning reagent. However, otoliths have internal layering (18–20) that might make them vulnerable to impregnation with diagenetic fluids, especially early in the diagenetic history. This motivated us to examine the effectiveness of cleaning whole otoliths *vs*. powdered otoliths. Secondly, we also examined the necessity of a secondary cleaning after possible exposure to contaminants from sample handling such as microscopic imaging and the powdering process (Fig. S3B). Finally, sagittal otoliths are typically precipitated as aragonite (8, 21, 22). Aragonite is particularly vulnerable to recrystallization and diagenetic alteration, which is a concern in otolith paleontology, both with regard to taxonomy and the preservation of calcium carbonate isotopic and elemental composition (23–27). This concern regarding otolith aragonite preservation motivated our investigations of whether preservation state showed signs of impacting $\delta^{15}N_{oto}$ (Table S2, Fig. S4).

We did not observe different patterns in N content or $\delta^{15}N_{oto}$ by cleaning category or sample form, which, in isolation, suggests that the initial cleaning of whole otoliths was sufficiently intensive for consistent N content and $\delta^{15}N_{oto}$ data. The one modest exception is that poorly preserved otoliths, when cleaned as powders, yielded lower N content compared to whole poorly-preserved otoliths and to well preserved otoliths at either cleaning level or in either form (Table S2). These data leave open the possibility that a small portion of the organic matter in poorly preserved otoliths may have been redistributed within the otolith interior, making it more susceptible to removal by grinding and oxidative cleaning. Although poorly preserved, powdered otoliths also appear to have slightly lower $\delta^{15}N_{oto}$ in our cleaning test, the lower N content and difference in $\delta^{15}N_{oto}$ observed may be explained by inter-otolith variation. We found that $\delta^{15}N_{oto}$ with *vs*. without cleaning after powdering falls closely on the 1:1 line (Fig. S5), implying that the effect of the cleaning of the powder is minor relative to natural $\delta^{15}N_{oto}$ variations within a given fish population. More broadly, there is no significant correlation between $\delta^{15}N_{oto}$ and N content ($R^2<0.05$, Fig. S6), which suggests that the organics that were removed were isotopically similar to those that remained internal to the otolith grains after powdering and cleaning. The results also imply that neither sample handling (imaging and powdering) nor an additional cleaning step impact $\delta^{15}N_{oto}$. Partial removal of a N pool with the typical preference for ^{14}N causes sample $\delta^{15}N$ to rise as the N content declines (28, 29). The lack of the correlation between $\delta^{15}N_{oto}$ and N content of poorly preserved otoliths argues against such diagenetic processes as a major influence on $\delta^{15}N_{oto}$ in these ~65-85 Myr old otoliths (Fig. S6).

The consistent $\delta^{15}N_{oto}$ across sample form (whole *vs*. powdered otolith) and cleaning methods implies one of the following. First, the otolith interiors may have never

had significant exposure to diagenetic fluids, such that all internal organic matter is original to the formation of the otolith. For example, otolith-bound N may be dominated by "mineral-bound" N that is either intra-crystalline or occurs at inaccessible boundaries between crystals (30). The FTIR indicated aragonitic composition in our fossil otoliths, even for poorly preserved otoliths, such that diagenetic alteration may not have had the opportunity to redistribute the organic matter that was biomineral-bound in the original otolith (Fig. S8 and S9). Second, any porosity in the otolith aragonite matrix may be penetrated by the cleaning solution, such that any organic matter that was not strictly biomineral-bound is removed completely (and/or without fractionation).

The results of examining the form of the sample (whole *vs*. powdered), preservation status (good *vs*. poor), and cleaning level (initial *vs*. initial + secondary) support the following conclusions: (1) Our imaging and grinding procedures rarely introduce organic contaminants; (2) An additional bleach cleaning is not necessary to obtain robust $\delta^{15}N$ and N content data, but it also does not have any negative effect; and, (3) The cleaning is effective for both whole and powdered otoliths, regardless of the preservation status.

Erring on the side of caution, we advocate for crushing (e.g., powdering) otoliths prior to cleaning, especially when evaluation of the morphological features (Table S3, S4) suggests that the preservation of the otoliths is poor. For all of the fossil otoliths in this study, we first clean the whole otolith. The cleaned otoliths are then ground into powders and cleaned with a second bleach cleaning.

Possible Late Cretaceous ODZ change mechanisms. Based on comparisons with model simulations of global warming, previous studies have raised two sets of hypotheses for the

apparent negative correlation of ODZ volume and global temperature over the Cenozoic (31, 32), and we consider each of these for our Cretaceous data. The first set involves upper ocean processes, in particular, the potential for tropical productivity to decline under warm climates. While the location of ODZs is largely determined by thermocline circulation (33), they also rely on the tropical production fueled by wind-driven upwelling. Indeed, there is evidence that changes in wind-driven biological production led to ODZ changes over the last century (34). Cretaceous wind patterns and their changes in response to the climate are still unclear. However, during warming climate from Albian to Cenomanian-Turonian boundary, earlier in the Cretaceous, sedimentary records in the proto-Atlantic basin imply expanded Hadley Cells and weakened tropical trade winds (35). Therefore, the cooling from Campanian to Maastrichtian may have hosted a tightening of the Hadley Cell circulation and a strengthening of the trade winds (36), promoting equatorial upwelling and expansion of ODZs in the subsurface ocean.

A second set of hypothesized mechanisms for reducing suboxia under warming involves the ventilation of the deep ocean, the oxygen content of which can impact the ODZs through low latitude deep upwelling (37). However, Nd isotopic compositions in South Atlantic, Indian Ocean and the Southern Ocean point to the Southern Ocean as an important source for deep water throughout the Campanian-Maastrichtian interval (38), arguing against this as the origin of the Late Cretaceous ODZ change. The deep Pacific in the Late Cretaceous has been suggested to be similar to the modern deep Pacific, with most of its ventilation by the southern high latitudes (39, 40). Even in a modeling study that indicated North Pacific deep water production during the Cretaceous, it was maintained into the Late Cretaceous (41), arguing against changes in North Pacific deep water as a

cause for ODZ change in the Late Cretaceous. The modern ocean interior is also filled with North Atlantic Deep Water (NADW), and the global ocean oxygen content is sensitive to the relative importance of NADW and AABW in the global ocean interior (42). Specifically, a higher ratio of NADW to AABW lowers the mean oxygen content of the global ocean interior and in the North Pacific in particular (43–45). Initiation of NADW has been inferred to occur during the Maastrichtian (46), when the surface North Atlantic became warmer under a global cooling interval (47) and local $\delta^{13}C$ became more positive (48). A stronger connection between the North and South Atlantic has also been suggested by Nd isotopes to have developed ~69 Ma in the Maastrichtian, and this may have been due to initiation or strengthening of NADW formation (46). An initiation of NADW formation in the Maastrichtian would have lowered the mean ocean oxygenation, and thus may have contributed to strengthening the ODZs, potentially explaining the Campanian-to-Maastrichtian increase in $\delta^{15}N_{oto}$.

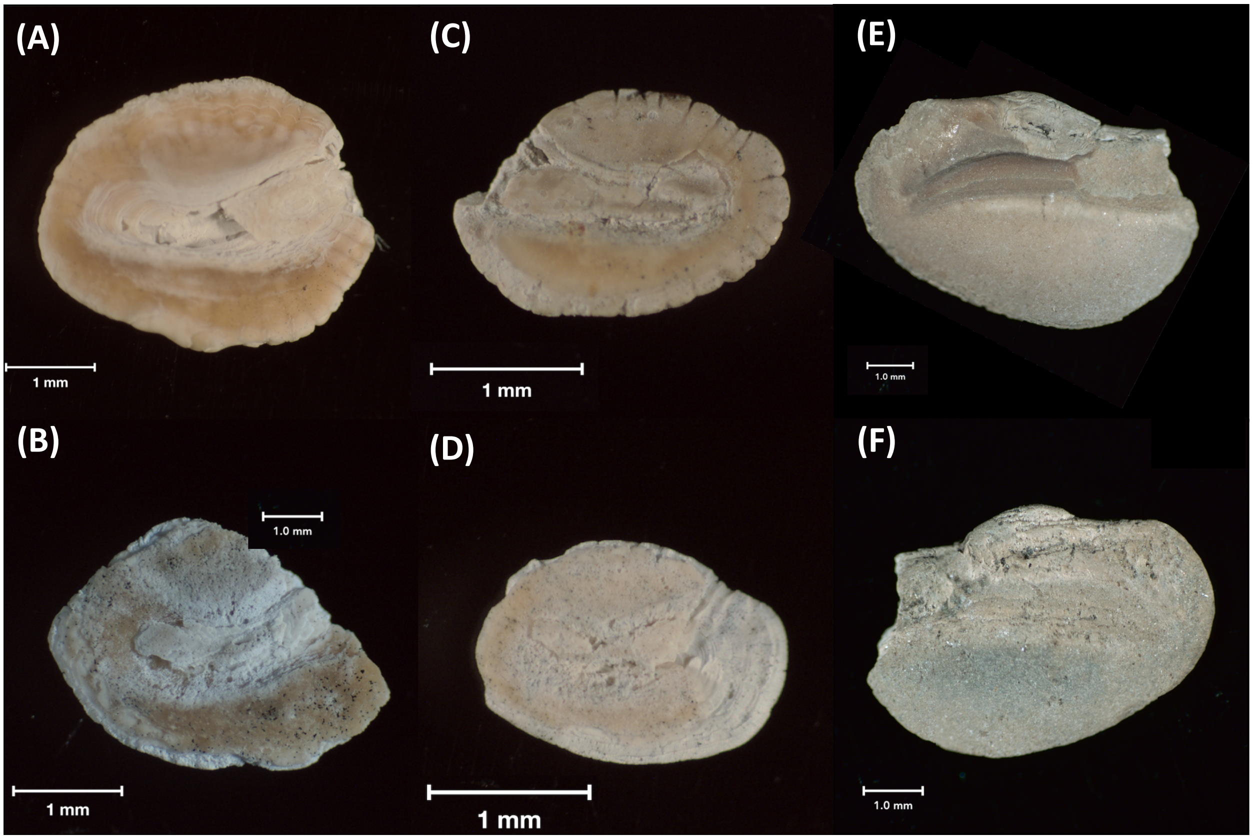

Figure S1. Stereo-microscope images of fossil otoliths. (A) Well preserved *E. maastrichtiensis* otolith; (B) poorly preserved *E. maastrichtiensis* otolith; (C) well preserved *E. zideki* otolith; (D) poorly preserved *E. zideki* otolith; (E-F) *Pterothrissus* sp. otoliths. These exemplary otoliths are from Severn Formation (A, D, E) and Tar Heel Formation (B, C, F), respectively.

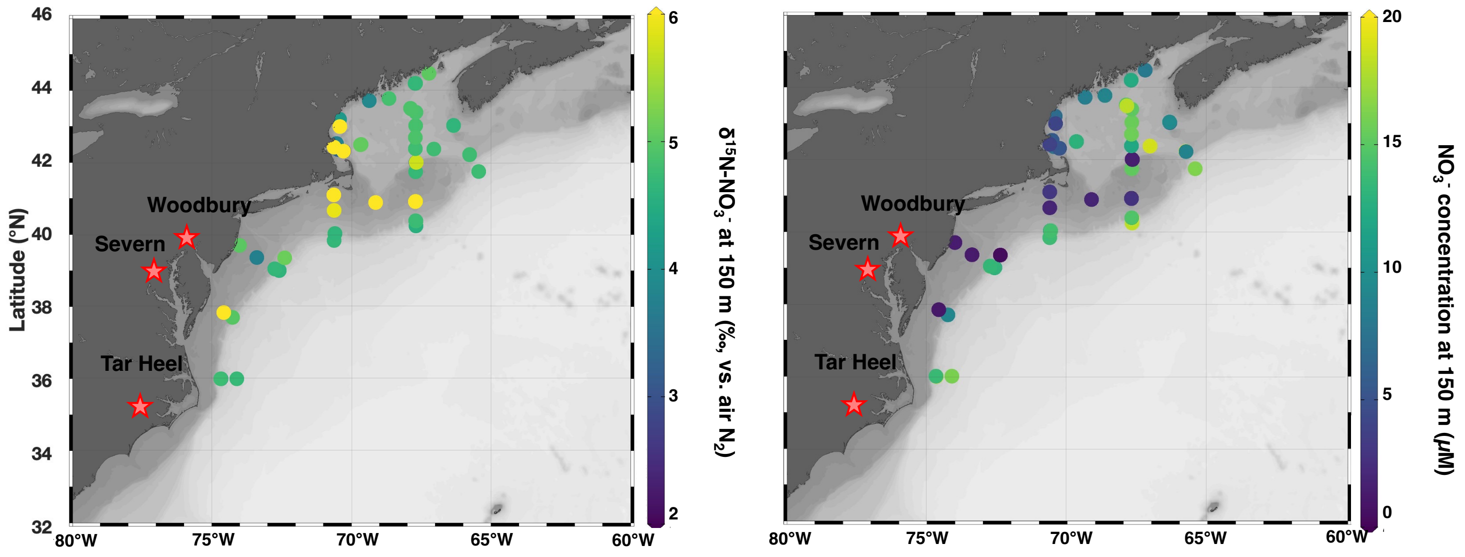

Figure S2. Nitrate $\delta^{15}N$ (left) and nitrate concentration (right) measurements at 150 m water depth from the western North Atlantic shelf system as colored circles, where the fossil otoliths were collected. Stars show the locations of three sampled Late Cretaceous formations, from north to south, the Woodbury Formation (New Jersey, Campanian), Severn Formation (Maryland, Maastrichtian) and Tar Heel Formation (North Carolina, Campanian). The map is generated with ODV (Schlitzer, Reiner, Ocean Data View, odv.awi.de, 2023).

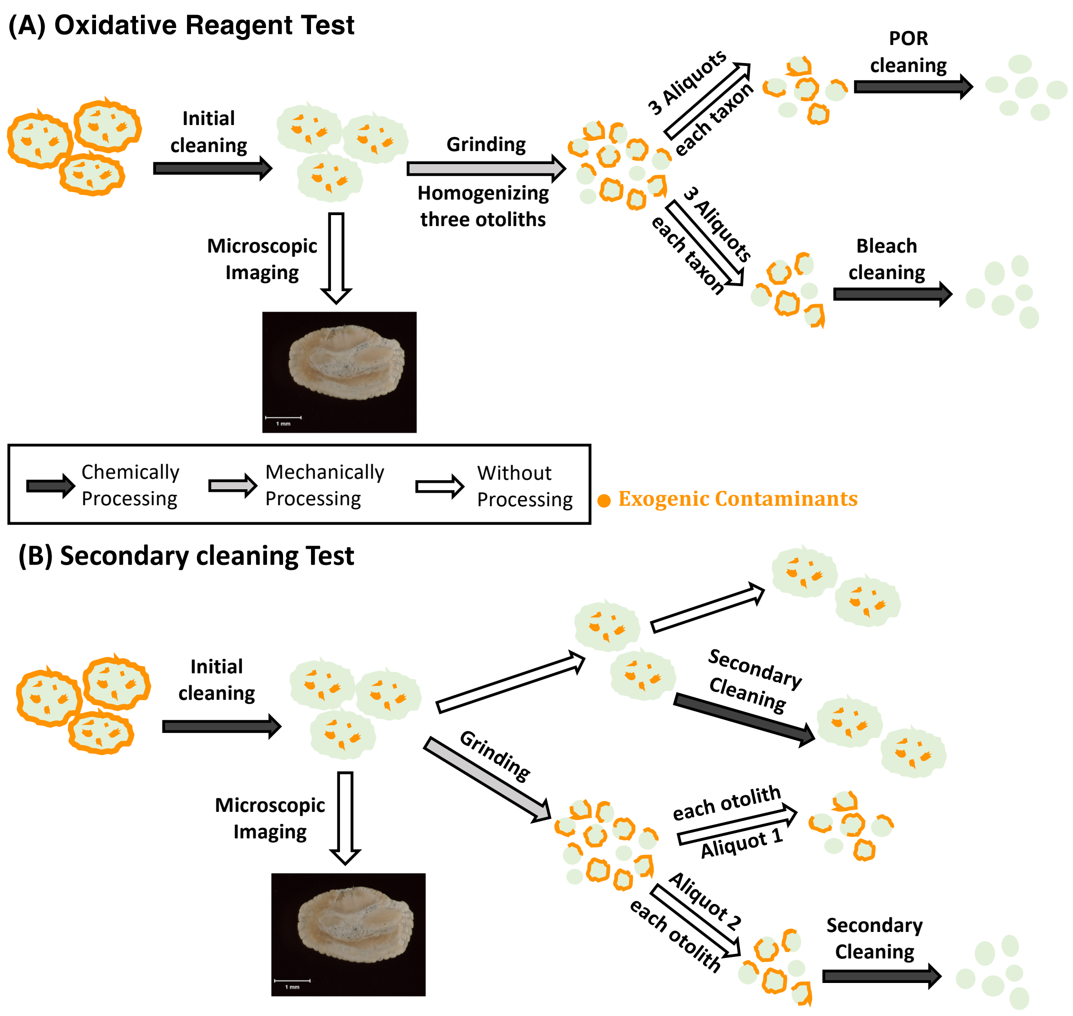

Figure S3. Illustration of the oxidative reagent test and secondary cleaning test performed on the Late Cretaceous otoliths. POR refers to the persulfate oxidative reagent, bleach refers to the 10-15% sodium hypochlorite solution. (A) Otoliths used in the oxidative reagent test are poorly preserved *E. maastrichtiensis* and *E. zideki* otoliths from Severn Formation. Initial cleaning includes three-step chemical cleaning with sodium polyphosphate, sodium hydrosulfite and potassium persulfate. (B) Secondary cleaning test is done on *E. maastrichtiensis* otoliths from Tar Heel Formation (North Carolina). The illustrated procedure is performed for a set of well-preserved otoliths and a set of poorly preserved otoliths.

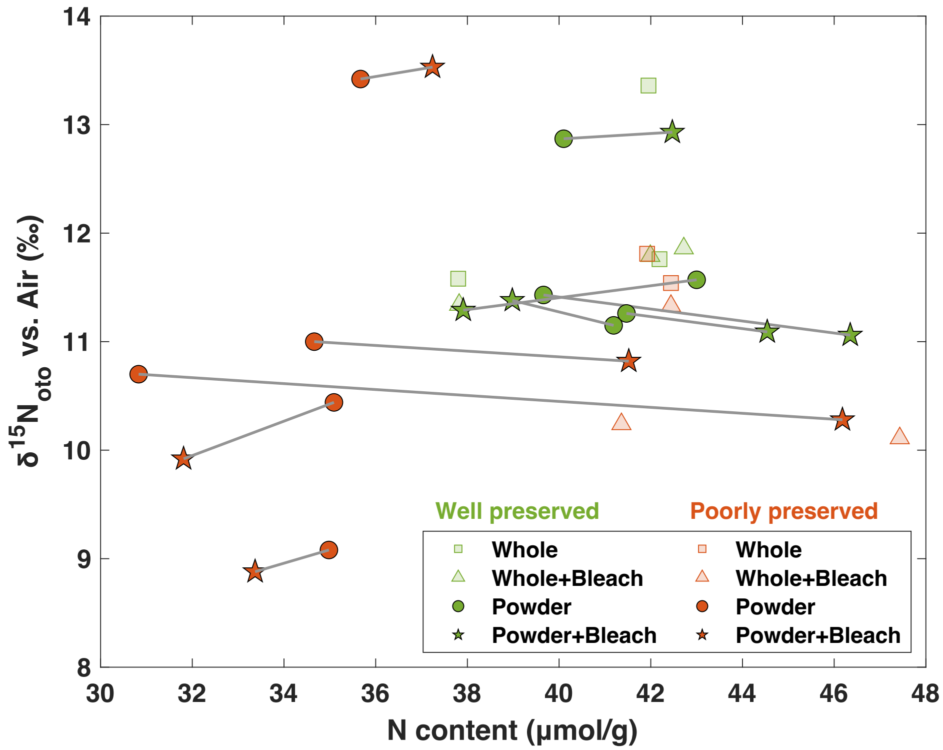

Figure S4. Otolith-bound $\delta^{15}N$ and N content cross plot. Green markers represent well preserved otoliths and orange markers represent the poorly preserved otoliths. Uncleaned powders are denoted in filled circles and cleaned powders are in stars. Measurements of the same otolith are connected with gray lines. Fainted symbols are measurements of individual whole otoliths. Squares are uncleaned whole otoliths, triangles are bleach-cleaned whole otoliths.

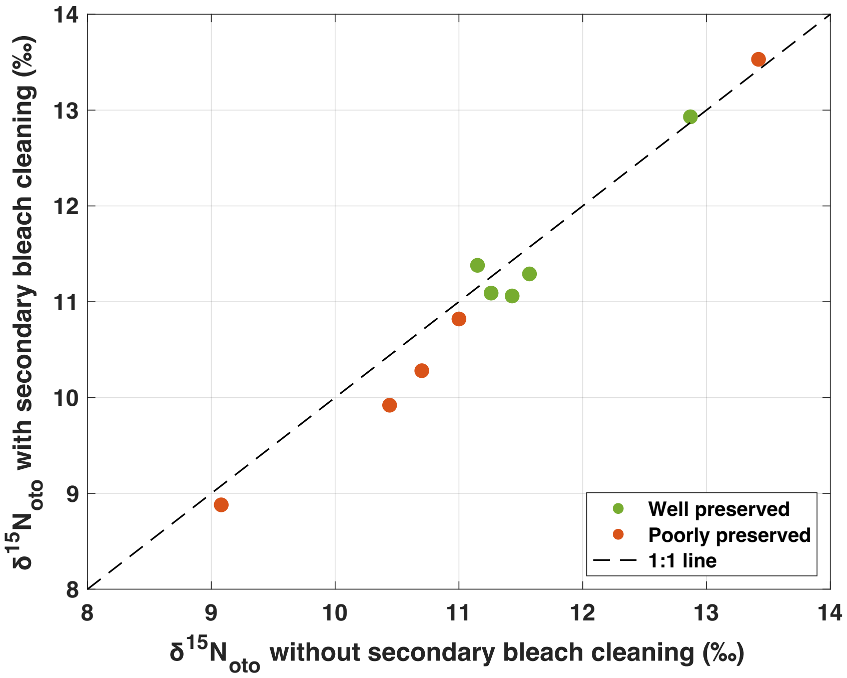

Figure S5. Otolith-bound $\delta^{15}N$ comparison of with and without secondary bleach cleaning. Measurements plotted are made with powdered otoliths. The green dots are from five well preserved otoliths and the orange dots are from five poorly preserved otoliths. The dashed 1:1 line indicates identical otolith-bound $\delta^{15}N$ regardless of the secondary bleach cleaning.

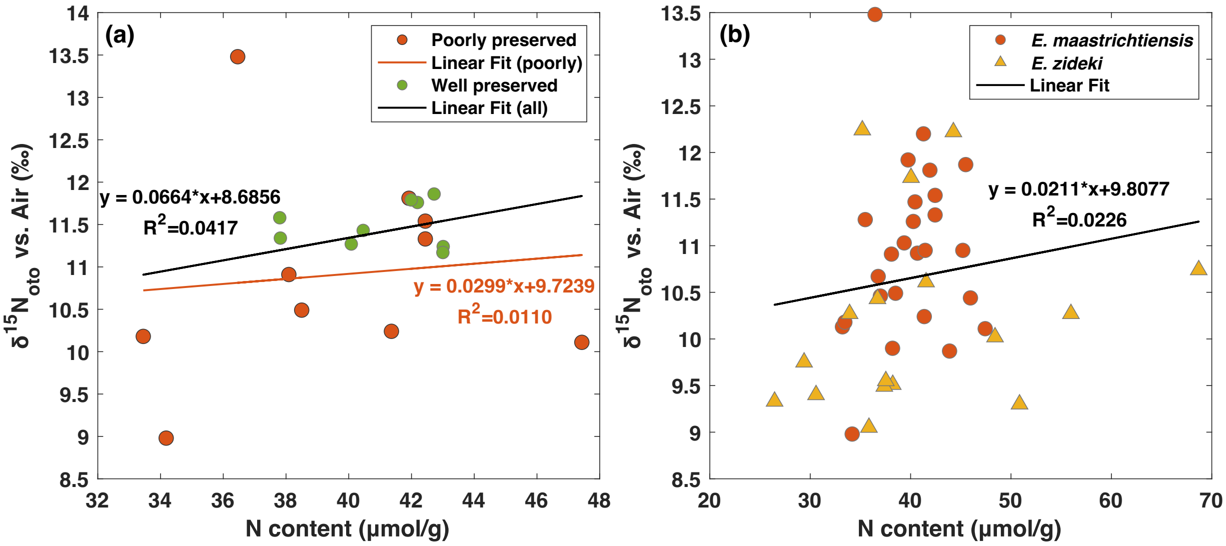

Figure S6. Correlation between otolith-bound $\delta^{15}N$ and N content. (a) Results from the secondary cleaning test. Well preserved otoliths are in green dots and poorly preserved otoliths are in orange. The orange line is regression from all results of poorly preserved otoliths, the black line is regressed from all data plotted here. (b) Results of all poorly preserved *Eutawichthys* spp. otoliths in this study. The *E. maastrichtiensis* are in orange dots and *E. zideki* are in yellow triangles.

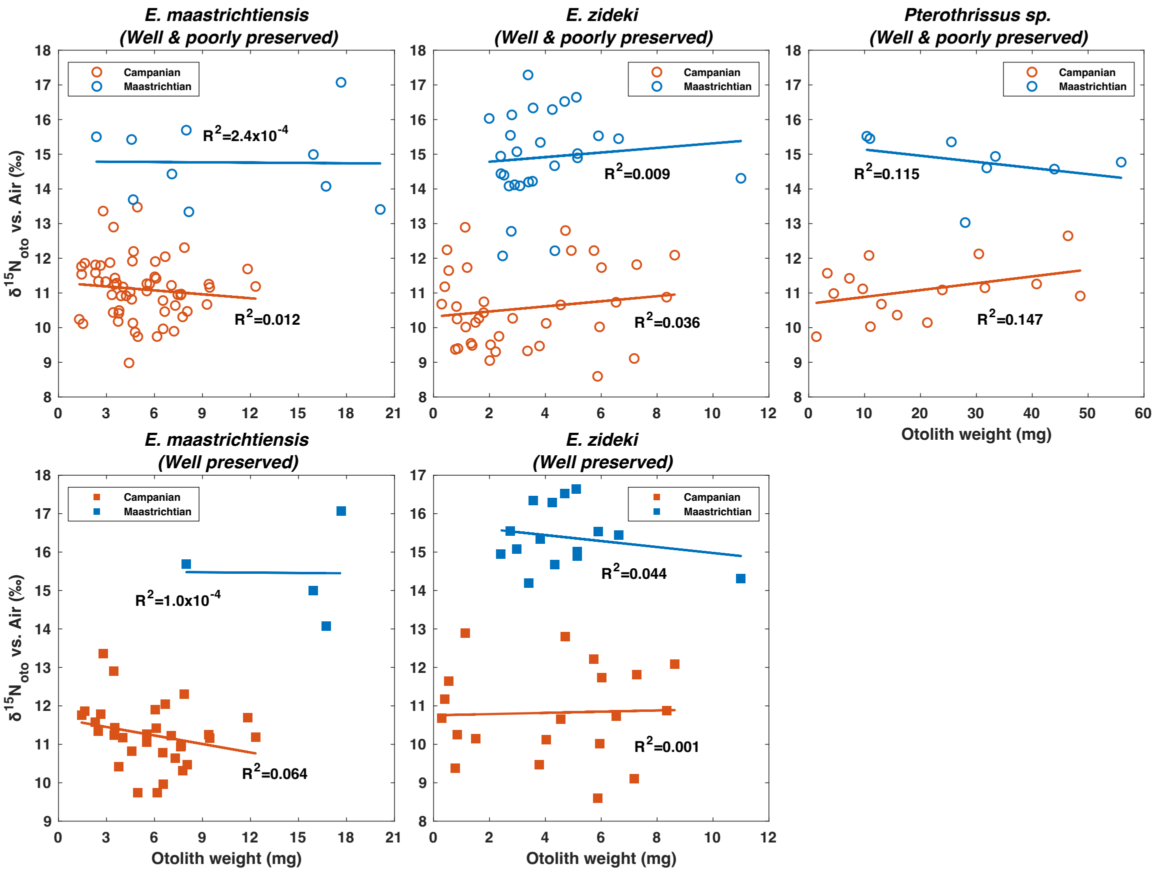

Figure S7. Correlation between otolith-bound $\delta^{15}N$ and otolith weight. Upper panels from left to right show all the individual otolith measurements of *E. maastrichtiensis*, *E. zideki* and *Pterothrissus* sp.; lower panels show well preserved otolith measurements only. Orange markers indicate otoliths in Campanian age, and blue markers indicate otoliths in Maastrichtian age. The lines are fitted linear regression and the color corresponds to each geologic interval (orange = Campanian; blue = Maastrichtian).

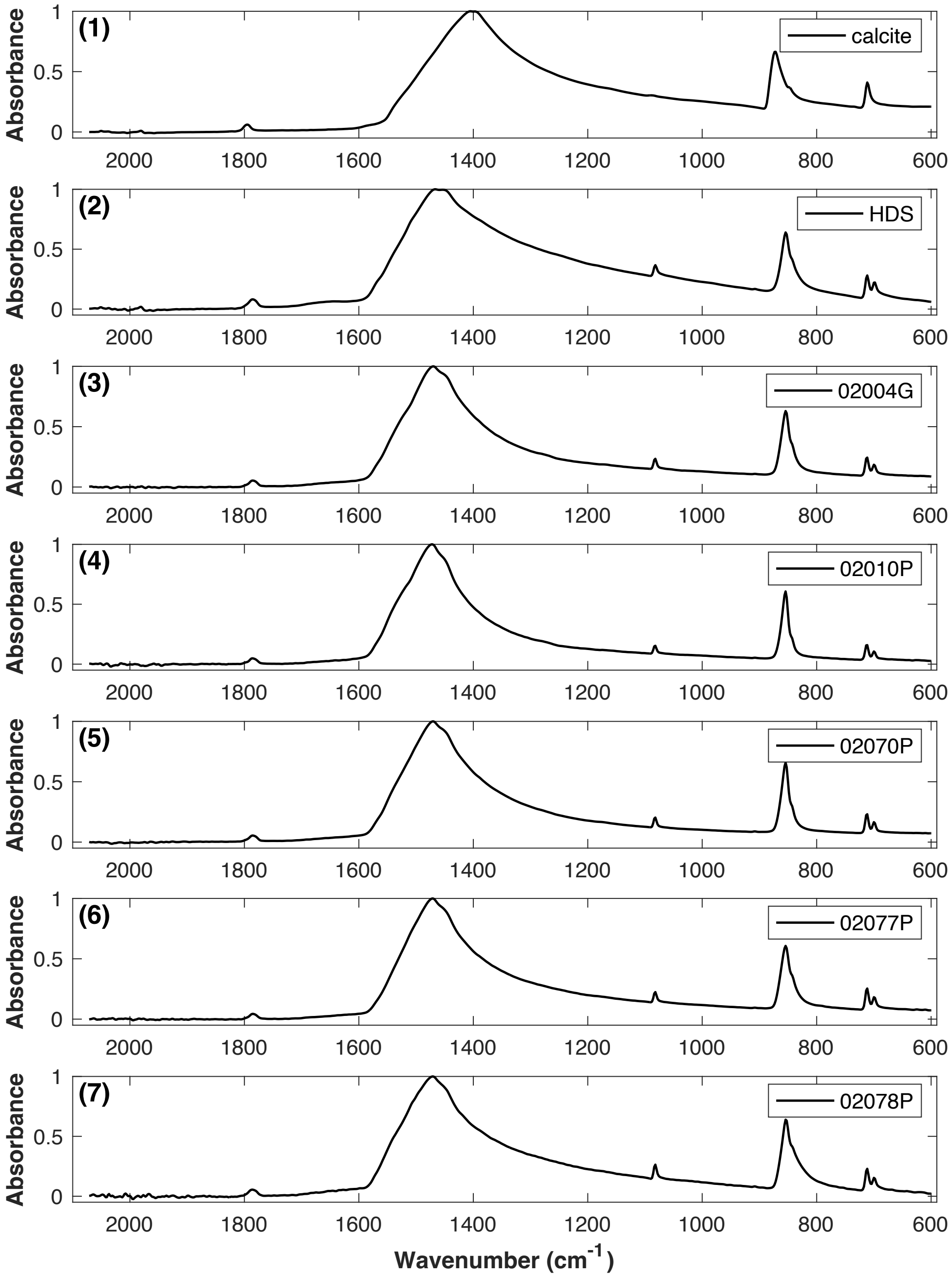

Figure S8. FTIR mineralogy of fossil E. zideki otoliths. (Panels 1-2) are from standard materials of calcite and in-house otolith standard (HDS, aragonite). (Panels 3-4) Well preserved otolith (panel 3) and poorly preserved otolith (panel 4) from Woodbury Formation in New Jersey, U.S.A. (Panels 5-7) Poorly preserved otoliths from Severn Formation in Maryland, U.S.A.

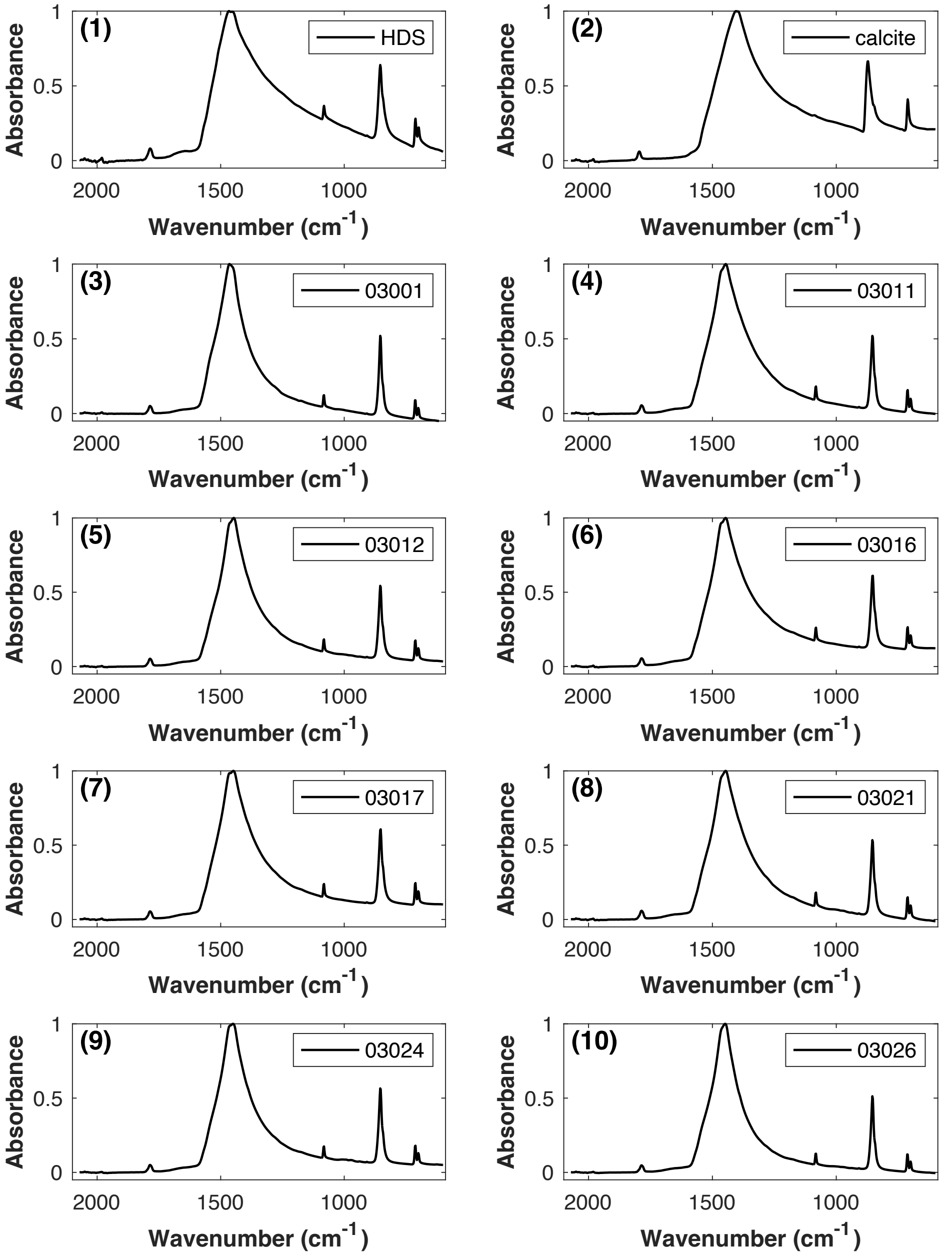

Figure S9. FTIR mineralogy of fossil *Pterothrissus* sp. otoliths. (Panels 1-2) are standards materials of otolith standard (HDS, aragonite) and calcite. Tested otoliths are from Woodbury Formation, New Jersey, U.S.A. (Panel 3), Tar Heel Formation, North Carolina, U.S.A. (Panels 4-7), and Severn Formation, Maryland, U.S.A. (Panel 8-10).

Table S1. Oxidative reagent test results of otolith-bound $\delta^{15}N$ and N content from Severn Formation otoliths.

**Species**	**$\Delta\delta_{B\text{-}P}$[#] (‰)**	**$\Delta N_{B\text{-}P}$[#] (μmol/g)**	$\frac{\Delta N_{B-P}}{Avg(N)}$	**Oxidative reagent**	**n[$]**	**$\delta^{15}N_{oto}$ (‰)**	**N content (μmol/g)**
Eutawichthys maastrichtiensis[1]	0.32[*] (p=0.0532)	0.32[*] (p=0.0532)	0.66%[*]	Bleach	3	13.83 ± 0.14	32.74 ± 0.49
				POR	3	13.51 ± 0.14	32.52 ± 1.04
Eutawichthys zideki[2]	0.22[*] (p=0.2533)	1.06[*] (p=0.0532)	3.58%[*]	Bleach	3	15.54 ± 0.27	30.09 ± 1.04
				POR	3	15.32 ± 0.10	29.03 ± 1.26

[1]Two whole otoliths were superficially cleaned and ground to yield one homogenized powdered sample.
[2]Five whole otoliths were superficially cleaned and ground to yield one homogenized powdered sample.
[*]Offsets are insignificantly different in Student's t-test (95% significance level).
[#]$\Delta\delta_{B\text{-}P}$ and $\Delta N_{B\text{-}P}$ refer to $\delta^{15}N$ and N content difference between bleach and POR cleaning of the powdered sample.
[$]The n refers to replicates of oxidative cleaning and analysis, all starting from a single homogenous powdered sample.

Table S2. Secondary cleaning test results of otolith-bound $\delta^{15}N$ and N content from different cleaning treatments.

Cleaning Regime	Preservation	Secondary cleaning	Number of otoliths	$\delta^{15}N_{oto}$ (‰)	N content (μmol/g)
Whole	Good	No	3*	12.24 ± 0.98	40.65 ± 2.47
		Yes	3*	11.66 ± 0.28	40.85 ± 2.64
	Poor	No	2*	11.67 ± 0.19	42.18 ± 0.36
		Yes	3*	10.56 ± 0.67	43.74 ± 3.24
Powder	Good	No	5#	11.66 ± 0.70	41.08 ± 1.31
		Yes	5#	11.55 ± 0.78	42.05 ± 3.59
	Poor	No	5&	10.93 ± 1.58	34.25 ± 1.94
		Yes	5&	10.69 ± 1.74	38.02 ± 5.91

*Each otolith was individually processed and analyzed.
#&Each otolith was individually ground and divided into two aliquots for analysis with and without secondary cleaning.

Table S3. Key morphological features to determine the preservation state of otoliths of *E. maastrichtiensis*.

No.	Morphological feature	Present? Yes/no
1.	Specimen is primarily complete with no major breaks, erosion, or leaching. Can be determined by referring to figures of complete specimens)*	
2.	Sulcus is present and obvious with the ostium and cauda clearly defined on the inner face of the otolith specimen.	
3.	The ostium of the otolith specimen is curved strongly upwards toward the anterior or anterodorsal margin.	
4.	There is clearly defined colliculum in the ostium that is much larger than the colliculum of the cauda in the otolith specimen.	
5.	The cauda is approximately the same length as the length of the ostium of the otolith specimen.	
6.	The cauda of the otolith specimen is curved strongly upwards toward the posterior or posterodorsal margin.	
7.	An obvious, well-developed crista superior is present just above the sulcus on the otolith specimen.	
8.	A narrow, somewhat oblong-shaped dorsal depression is visible above the sulcus on the otolith specimen.	
9.	The ventral furrow, which is situated approximately half-way between the ventral margin of the sulcus and ventral margin, is visible on the otolith.	
10.	A blunt, slightly supramedian rostrum with a small antirostrum is present on the anterior margin of the otolith specimen.	

*Figures of complete specimens may be found in Nolf and Stringer (1996), Nolf (2013), Stringer et al. (2020), Schwarzhans and Stringer (2020), and Stringer and Schwarzhans (2021).

Table S4. Key morphological features to determine the preservation state of otoliths of *E. zideki*.

No.	Morphological feature	Present? Yes/no
1.	Specimen is primarily complete with no major breaks, erosion, or leaching. Can be determined by referring to figures of complete specimens)*	
2.	Sulcus is present and obvious with the ostium and cauda clearly defined on the inner face of the otolith specimen.	
3.	The ostium of the otolith specimen is curved slightly upwards toward the anterior or anterodorsal margin.	
4.	There is clearly defined colliculum in the ostium that is larger than the colliculum of the cauda in the otolith specimen.	
5.	The cauda is slightly less in length than that of the ostium of the otolith specimen.	
6.	The cauda of the otolith specimen shows very little or no curvature toward the posterior or posterodorsal margin.	
7.	An obvious, well-developed crista superior is present above the sulcus on the otolith specimen.	
8.	A narrow, somewhat linear-shaped dorsal depression is visible above the sulcus on the otolith specimen.	
9.	The ventral furrow, which is situated close the ventral margin is present and visible on the otolith specimen.	
10.	A broad, median rostrum with a blunt tip is present on the anterior margin of the otolith specimen.	

*Figures of complete specimens may be found in Nolf and Stringer (1996), Nolf (2013), Stringer et al. (2020), Schwarzhans and Stringer (2020), and Stringer and Schwarzhans (2021).

Dataset S1. Late Cretaceous fossil otolith-bound $\delta^{15}N$ ($\delta^{15}N_{oto}$) data, including the results from the oxidative reagent test and the secondary cleaning test, and $\delta^{15}N_{oto}$ results of all fossil otoliths analyzed in this study.

Dataset S2. U.S. Northeast oceanographic $\delta^{15}N$ data from the seawater nitrate, suspended PN, zooplankton, and modern otoliths of butterfish.

SI References

1. D. Marconi, *et al.*, Nitrate isotope distributions on the US GEOTRACES North Atlantic cross-basin section: Signals of polar nitrate sources and low latitude nitrogen cycling. *Marine Chemistry* **177**, 143–156 (2015).

2. N. Van Oostende, *et al.*, Variation of summer phytoplankton community composition and its relationship to nitrate and regenerated nitrogen assimilation across the North Atlantic Ocean. *Deep Sea Research Part I: Oceanographic Research Papers* **121**, 79–94 (2017).

3. X. Peng, *et al.*, Nitrogen uptake and nitrification in the subarctic North Atlantic Ocean. *Limnology and Oceanography* **63**, 1462–1487 (2018).

4. P. J. Politis, J. K. Galbraith, P. Kostovick, R. W. Brown, Northeast Fisheries Science Center bottom trawl survey protocols for the NOAA Ship Henry B. Bigelow. *Northeast Fisheries Science Center Reference Document 14-06* (2014). https://doi.org/10.7289/V5C53HVS.

5. M. A. Weigand, J. Foriel, B. Barnett, S. Oleynik, D. M. Sigman, Updates to instrumentation and protocols for isotopic analysis of nitrate by the denitrifier method. *Rapid Commun. Mass Spectrom.* **30**, 1365–1383 (2016).

6. F. Nydahl, On the peroxodisulphate oxidation of total nitrogen in waters to nitrate. *Water Research* **12**, 1123–1130 (1978).

7. A. Martínez-García, *et al.*, Laboratory Assessment of the Impact of Chemical Oxidation, Mineral Dissolution, and Heating on the Nitrogen Isotopic Composition of Fossil-Bound Organic Matter. *Geochem Geophys Geosyst* **23** (2022).

8. J. A. Lueders-Dumont, X. T. Wang, O. P. Jensen, D. M. Sigman, B. B. Ward, Nitrogen isotopic analysis of carbonate-bound organic matter in modern and fossil fish otoliths. *Geochimica et Cosmochimica Acta* **224**, 200–222 (2018).

9. E. Real, *et al.*, Growth patterns of the lanternfish Ceratoscopelus maderensis in the western Mediterranean Sea. *Scientia Marina* **85**, 71–80 (2021).

10. G. Stringer, W. Schwarzhans, Upper Cretaceous teleostean otoliths from the Severn Formation (Maastrichtian) of Maryland, USA, with an unusual occurrence of Siluriformes and Beryciformes and the oldest Atlantic coast Gadiformes. *Cretaceous Research* **125**, 104867 (2021).

11. S. Ramsvatn, T. Pedersen, Ontogenetic niche changes in haddock Melanogrammus aeglefinus reflected by stable isotope signatures, δ13C and δ15N. *Mar. Ecol. Prog. Ser.* **451**, 175–185 (2012).

12. O. T. Albert, Ecology of haddock (Melanogrammus aeglefinus L.) in the Norwegian Deep. *ICES Journal of Marine Science* **51**, 31–44 (1994).

13. A. N. Knapp, D. M. Sigman, F. Lipschultz, N isotopic composition of dissolved organic nitrogen and nitrate at the Bermuda Atlantic Time-series Study site. *Global Biogeochemical Cycles* **19** (2005).

14. G. L. Stringer, Evidence and implications of marine invertebrate settlement on Eocene otoliths from the Moodys Branch Formation of Montgomery Landing (Louisiana, U.S.A.). *Cainozoic Research* **16**, 3–12 (2016).

15. G. L. Stringer, L. D. Oman, R. F. Badger, Woodbury Formation (Campanian) in New Jersey yields largest known Cretaceous otolith assemblage of teleostean fishes in North America. *Proceedings of the Academy of Natural Sciences of Philadelphia* **165**, 15–36 (2016).

16. H. Ren, *et al.*, Foraminiferal Isotope Evidence of Reduced Nitrogen Fixation in the Ice Age Atlantic Ocean. *Science* **323**, 244–248 (2009).

17. B. Pokroy, *et al.*, Anisotropic lattice distortions in the mollusk-made aragonite: A widespread phenomenon. *Journal of Structural Biology* **153**, 145–150 (2006).

18. R. Fablet, *et al.*, Shedding Light on Fish Otolith Biomineralization Using a Bioenergetic Approach. *PLOS ONE* **6**, e27055 (2011).

19. T. Schulz-Mirbach, A. Götz, E. Griesshaber, M. Plath, W. W. Schmahl, Texture and nano-scale internal microstructure of otoliths in the Atlantic molly, Poecilia mexicana: A high-resolution EBSD study. *Micron* **51**, 60–69 (2013).

20. D. Nolf, *The diversity of fish otoliths, past and present*, E. Steurbaut, R. Brzobohatý, K. Hoedemakers, Eds. (Royal Belgian Institute of Natural Sciences, 2013).

21. D. Carlström, A Crystallographic Study of Vertebrate Otoliths. *Biological Bulletin* **125**, 441–463 (1963).

22. S. Campana, Chemistry and composition of fish otoliths:pathways, mechanisms and applications. *Mar. Ecol. Prog. Ser.* **188**, 263–297 (1999).

23. W. P. Patterson, Oldest isotopically characterized fish otoliths provide insight to Jurassic continental climate of Europe. *Geol* **27**, 199 (1999).

24. G. D. Price, D. Wilkinson, M. B. Hart, K. N. Page, S. T. Grimes, Isotopic analysis of coexisting Late Jurassic fish otoliths and molluscs: Implications for upper-ocean water temperature estimates. *Geology* **37**, 215–218 (2009).

25. A. Woydack, B. Morales-Nin, Growth patterns and biological information in fossil fish otoliths. *Paleobiology* **27**, 369–378 (2001).

26. D. Vanhove, P. Stassen, R. P. Speijer, E. Steurbaut, Assessing paleotemperature and seaonality during the early eocene climatic optimum (EECO) in the Belgian Basin

by means of fish otolith stable O and C isotopes. *Geologica belgica* **14**, 143–158 (2011).

27. D. Vanhove, P. Stassen, R. P. Speijer, P. Claeys, E. Steurbaut, Intra- and intertaxon stable O and C isotope variability of fossil fish otoliths: an early Eocene test case. *Austrian Journal of Earth Sciences* **105**, 200–207 (2012).

28. J. Möbius, N. Lahajnar, K.-C. Emeis, Diagenetic control of nitrogen isotope ratios in Holocene sapropels and recent sediments from the Eastern Mediterranean Sea. *Biogeosciences* **7**, 3901–3914 (2010).

29. R. S. Robinson, *et al.*, A review of nitrogen isotopic alteration in marine sediments. *Paleoceanography* **27** (2012).

30. J. A. Lueders-Dumont, *et al.*, Controls on the nitrogen isotopic composition of fish otolith organic matter: Lessons from a controlled diet switch experiment. *Geochimica et Cosmochimica Acta* **316**, 69–86 (2022).

31. A. Auderset, *et al.*, Enhanced ocean oxygenation during Cenozoic warm periods. *Nature* **609**, 77–82 (2022).

32. A. V. Hess, *et al.*, A well-oxygenated eastern tropical Pacific during the warm Miocene. *Nature* 1–5 (2023). https://doi.org/10.1038/s41586-023-06104-6.

33. J. R. Luyten, J. Pedlosky, H. Stommel, The Ventilated Thermocline. *Journal of Physical Oceanography* **13**, 292–309 (1983).

34. C. Deutsch, *et al.*, Centennial changes in North Pacific anoxia linked to tropical trade winds. *Science* **345**, 665–668 (2014).

35. T. Wagner, P. Hofmann, S. Flögel, Marine black shale deposition and Hadley Cell dynamics: A conceptual framework for the Cretaceous Atlantic Ocean. *Marine and Petroleum Geology* **43**, 222–238 (2013).

36. J. Lu, G. A. Vecchi, T. Reichler, Expansion of the Hadley cell under global warming. *Geophysical Research Letters* **34** (2007).

37. A. Gnanadesikan, J. P. Dunne, J. John, Understanding why the volume of suboxic waters does not increase over centuries of global warming in an Earth System Model. *Biogeosciences* **9**, 1159–1172 (2012).

38. S. A. Robinson, D. P. Murphy, D. Vance, D. J. Thomas, Formation of "Southern Component Water" in the Late Cretaceous: Evidence from Nd-isotopes. *Geology* **38**, 871–874 (2010).

39. S. J. Haynes, *et al.*, Constraining sources and relative flow rates of bottom waters in the Late Cretaceous Pacific Ocean. *Geology* **48**, 509–513 (2020).

40. C. Jung, S. Voigt, O. Friedrich, M. C. Koch, M. Frank, Campanian-Maastrichtian ocean circulation in the tropical Pacific. *Paleoceanography* **28**, 562–573 (2013).

41. Y. Donnadieu, E. Pucéat, M. Moiroud, F. Guillocheau, J.-F. Deconinck, A better-ventilated ocean triggered by Late Cretaceous changes in continental configuration. *Nat Commun* **7**, 10316 (2016).

42. J. R. Toggweiler, R. Murnane, S. Carson, A. Gnanadesikan, J. L. Sarmiento, Representation of the carbon cycle in box models and GCMs, 2, Organic pump. *Global Biogeochemical Cycles* **17** (2003).

43. M. P. Hain, D. M. Sigman, G. H. Haug, "The Biological Pump in the Past" in *Treatise on Geochemistry*, (Elsevier, 2014), pp. 485–517.

44. I. Marinov, M. Follows, A. Gnanadesikan, J. L. Sarmiento, R. D. Slater, How does ocean biology affect atmospheric pCO2? Theory and models. *Journal of Geophysical Research: Oceans* **113** (2008).

45. D. M. Sigman, M. P. Hain, G. H. Haug, The polar ocean and glacial cycles in atmospheric CO2 concentration. *Nature* **466**, 47–55 (2010).

46. K. G. MacLeod, C. Isaza Londoño, E. E. Martin, Á. Jiménez Berrocoso, C. Basak, Changes in North Atlantic circulation at the end of the Cretaceous greenhouse interval. *Nature Geosci* **4**, 779–782 (2011).

47. K. G. MacLeod, B. T. Huber, C. Isaza-Londoño, North Atlantic warming during global cooling at the end of the Cretaceous. *Geol* **33**, 437 (2005).

48. T. D. Frank, M. A. Arthur, Tectonic forcings of Maastrichtian ocean-climate evolution. *Paleoceanography* **14**, 103–117 (1999).
